# The Internal–External Locus of Control Short Scale–4 (IE-4): A comprehensive validation of the English-language adaptation

**DOI:** 10.1371/journal.pone.0271289

**Published:** 2022-07-11

**Authors:** Désirée Nießen, Isabelle Schmidt, Katharina Groskurth, Beatrice Rammstedt, Clemens M. Lechner

**Affiliations:** GESIS–Leibniz Institute for the Social Sciences, Mannheim, Germany; Public Library of Science, UNITED KINGDOM

## Abstract

The Internal–External Locus of Control Short Scale–4 (IE-4) measures two dimensions of the personality trait locus of control with two items each. IE-4 was originally developed and validated in German and later translated into English. In the present study, we assessed the psychometric properties (i.e., objectivity, reliability, validity) of the English-language IE-4, compared these psychometric properties with those of the German-language source version, and tested measurement invariance across both language versions. Using heterogeneous quota samples from the UK and Germany, we find that the English-language adaptation has satisfactory reliability and plausible correlations with 11 external variables (e.g., general self-efficacy, self-esteem, impulsive behavior, Emotional Stability), which are comparable with those of the German-language source version. Moreover, metric measurement invariance of the scale holds when comparing the UK and Germany, implying the comparability of correlations based on the latent factors across the two nations. As an ultra-short scale (completion time < 30 s), IE-4 lends itself particularly to the assessment of locus of control in survey contexts in which assessment time or questionnaire space are limited. It can be applied in a variety of research disciplines, such as psychology, sociology, or economics.

## Introduction

Locus of control is defined as a generalized expectation of internal or external control of reinforcement [1]. Individuals with an internal locus of control generally believe that events are contingent upon their own actions. By contrast, individuals with an external locus of control generally perceive events to be “the result of luck, chance, fate”, or “as under the control of powerful others” [1, p. 1] (see also [2]).

Internal and external locus of control are predictive of a variety of behavioral, cognitive, and affective outcomes in different areas of life, including well-being, satisfaction, and performance-related behavior and outcomes [3]. Studies investigating internal and external locus of control need a valid and economical measure of these dispositions, particularly in research settings with severe time limitations or other constraints on questionnaire length. Motivated by this need in the German-language context, Kovaleva et al. [4] developed the Internale-Externale-Kontrollüberzeugung-4 (IE-4), an ultra-short scale measuring internal and external locus of control with two items each. IE-4 is a highly economic scale with a completion time of about 30 s (estimated value; based on own experiences, the average completion time for one personality item typically ranges between 5 and 8 s). The scale was validated in a large and diverse random sample of adults in Germany. In the absence of a comparable ultra-short scale for the measurement of internal and external locus of control in an English-language context, Kovaleva et al. [4] translated and adapted IE-4 to English. We named this English-language version Internal–External Locus of Control Short Scale–4 (IE-4).

An empirical validation of the English-language IE-4 was hitherto lacking. The aim of the present study is to fill this gap. Specifically, first, we analyzed the reliability and factorial structure of the scale, and its correlations with a broad range of 11 external correlates in order to validate the scale and to embed it in a nomological network. Second, we compared these psychometric properties with those of the German-language source version. And finally, we investigated measurement invariance across the two language versions. For this purpose, we used heterogeneous quota samples of adults from the United Kingdom (UK) and Germany (DE).

## Theoretical background

The theoretical background section of this paper is based on, and parts of it are taken from, Kovaleva et al. [4]. The concept of the locus of control of reinforcement (i.e., rewards or punishments) was developed in the 1950s by Julian Rotter within the framework of social learning theory [1, 5]. Locus of control describes the extent to which individuals believe that rewards or failures in life are contingent upon their own actions or are controlled by external forces. Rotter [1, 5] conceptualized locus of control as a unidimensional continuum, with an extreme expression of internal locus of control beliefs at one pole and an extreme expression of external locus of control beliefs at the other. Individuals who have a more internal locus of control generally believe that events are under their own control, whereas individuals who have a more external locus of control generally believe that their lives are under the control of powerful others or fate [1].

In his social learning theory, Rotter [1, 5] assumed that locus of control arose from an individual’s cognitions, perceptions, and learning within social situations. The experiences in these social situations are generalized to similar situations. As a result, a cross-contextual, stable locus of control evolves, which is conceptualized as a personality trait. Accordingly, locus of control maps onto the Big Five personality dimensions: A more internal locus of control is strongly related to higher levels of Emotional Stability (and also, but to a lesser degree, to higher Conscientiousness, Extraversion, Openness to Experience, and Agreeableness) [6].

Locus of control also explains several other phenomena within and across individuals, thereby rendering it an important construct for a variety of research questions. It has been related to a variety of behavioral [7], affective and cognitive [8], and physiological outcomes [9, 10] in different areas of life (e.g., health, education, work, or social relationships), across different age groups [11, 12], and across countries [13].

For instance, a plethora of research indicates that individuals who have a more internal locus of control spend more time on intellectual and academic activities compared with externally controlled individuals [7]. Consequently, internally controlled individuals tend to be more successful at school [14] and at work [3]. In a meta-analysis, Judge and Bono [15] found that a more internal locus of control was positively related to job satisfaction and job performance (see also [12]). Moreover, researchers have found significant positive relationships between internal locus of control and interpersonal trust [16, 17] as well as between internal locus of control and life satisfaction in different age groups [18, 19]. Furthermore, previous research has consistently reported that internal locus of control is strongly related to higher subjective well-being [13], general self-efficacy, and self-esteem [15]. In addition, because the constructs political efficacy and locus of control are based on internal and external control beliefs, researchers have found a positive association between internal locus of control and internal political efficacy [20, 21]. By contrast, external locus of control has been consistently reported to be negatively associated with life satisfaction [22], self-efficacy [23, 24], and self-esteem [25].

Regarding justice sensitivity, previous research has been reported the opposite direction compared to most other effects with locus of control, namely a negative correlation between internal locus of control and justice sensitivity as well as positive association between external locus of control and justice sensitivity [26, 27].

People with a more internal locus of control have been found to be more willing to take risks [28, 29], to show more impulsive behavior [30], and to have more optimistic expectations than externally controlled individuals who have been found to have more pessimistic expectations [31, 32]. They also seem to be more resilient: In a recent study with adults in the United States and five European countries (France, Germany, Italy, Spain, the United Kingdom), Sigurvinsdottir et al. [33] found that externally controlled individuals showed higher depression, anxiety, and stress across countries when confronted with the COVID-19 pandemic, compared with those individuals who were more internally controlled.

Locus of control has stimulated a lot of research since its initial introduction by Rotter [1, 5], and several measurement instruments have been developed for its assessment. Rotter [1] constructed the unidimensional 29-item Internal-External Locus of Control Scale (the I-E scale). However, the definition of internal‒external locus of control as a unidimensional, bipolar construct was theoretically and empirically challenged in the years that followed [2, 34]. Factorial analyses with Rotter’s I-E scale showed that at least two separate factors of internal and external locus of control should be extracted from it [2, 35] (but see Watson [36, p. 319], who concluded that “the identification of more than two factors should be done with considerable reluctance”). Levenson [2] developed the 24-item Internal, Powerful Others, and Chance Scale (IPC) to assess three dimensions in total. The first dimension relates to internal locus of control (I); the other two dimensions—powerful others orientation (P) and belief in chance (C)—relate to external locus of control. However, subsequent research failed to provide clear evidence for Levenson’s tripartite model or for other more recent structural models of locus of control [37] comprising multiple dimensions of internal and external locus of control [38].

Based on these findings, and aware of the general time constraints in surveys, Jakoby and Jacob [39] developed the German-language Kurzskalen zur Messung von Kontrollüberzeugungen [Short Scales for the Assessment of Locus of Control Orientations in Population Surveys] (KMKB), a two-dimensional 6-item scale measuring internal and external locus of control separately. These authors based their concept of external locus of control on Levenson’s definition [2], which comprised the dimensions powerful others orientation and belief in chance. Jakoby and Jakob [39] confirmed the two-factorial structure of internal‒external locus of control in a principal component analysis.

Given the lack of comprehensive validations of German-language locus of control scales such as the KMKB that were suitable for use in surveys, Kovaleva [40] and Kovaleva and colleagues [4] set out to construct and validate an economical locus of control scale for German-language survey contexts with severe time constraints. To measure internal‒external locus of control as a two-dimensional construct, they developed the Internale–Externale-Kontrollüberzeugung–4 (IE-4) scale, an ultra-short scale measuring the two dimensions with just two items each. Because a newly developed scale is only relevant if it is better than existing scales with regard to at least some quality criteria (e.g., more economical, higher construct validity, etc.), Kovaleva [40] compared the psychometric properties of the IE-4 scale with those of the KMKB scale. KMKB had hitherto been the only two-dimensional locus of control short scale usable in contemporary German-language (large-scale) surveys with known⸺that is, published⸺quality criteria, but these sufficient psychometric properties had never been replicated⸺or published⸺again outside the developers [40]. For another existing two-dimensional locus of control short scale, which was used in the German Socio-Economic Panel (SOEP), the psychometric properties have never been published [40]. Kovaleva [40] showed that, first, IE-4 measured the same two factors as KMKB; and, second, that both scales were sufficiently reliable and valid measures of locus of control. Because IE-4 has equally good psychometric properties as KMKB but is shorter (four vs. six items), IE-4 is more time-efficient and thus to be preferred [40].

### Development of the IE-4 scale

For the original German-language IE-4 scale, Kovaleva et al. [4] first developed 20 items based on the definition of internal and external locus of control proposed by Rotter [1]. In a second step, these items underwent cognitive pretesting to ensure item clarity and comprehensibility. Based on content-related aspects and factor-analytical investigations, four items were selected—two measuring internal and two measuring external locus of control (for more detailed information, see [40]; for the original German-language items, see S1 Appendix and [4, 40]). The German-language IE-4 scale was thoroughly validated based on a large and diverse random sample representative of the adult population in Germany in terms of age, gender, and educational attainment.

To enable social scientists to use IE-4 in an English-language context, the scale was adapted to the English language by Kovaleva et al. [4]. In a first step, the four items of IE-4 and their rating scale were translated into English following the TRAPD (Translation, Review, Adjudication, Pretesting, and Documentation) approach [41]. Two professional translators (English native speakers) translated the item wording and the response scale labels independently of each other into British English and American English, respectively. Second, an adjudication meeting was held, at which psychological experts, the two translators, and an expert in questionnaire translation reviewed the translation proposals and developed the final translation. The validation of the English-language version of IE-4 remained a desideratum until the present study.

The English-language items are displayed in Table 1 and in the S2 Appendix. As in the German-language source instrument, all items are positively worded in relation to the underlying constructs, internal and external locus of control. The items are answered using a 5-point rating scale ranging from *does not apply at all* (1) to *applies completely* (5). For each subscale, the unweighted mean score of the respective two items is computed to obtain subscale scores for internal and external locus of control. Computing a total mean score across both subscales is not recommended. We suggest that individual answers should be aggregated to the scale level only if there are no missing values on any of the two items. In our two samples (UK and Germany) there were no missing values. If there are missing values, we recommend using appropriate methods for handling missing data, such as multiple imputation [42] or full information maximum likelihood estimation (FIML) [43]. Furthermore, we do not recommend surveys with small sample sizes (e.g., total *N* ≤ 200–300) because small samples may cause technical problems and some estimates, such as standard errors of latent variables, may be inaccurate [44]. If users have only small samples available and want to compute a confirmatory factor analysis (CFA), they can fix the parameters (i.e., loadings and variances) of the model to the values from our study. In order to determine an appropriate sample size for the planned analyses, a power analysis can be carried out in advance.

**Table 1:** Wording of the English-language IE-4 items.

No.	Item	Subscale
1	I’m my own boss.	Internal
2	If I work hard, I will succeed.	Internal
3	Whether at work or in my private life: What I do is mainly determined by others.	External
4	Fate often gets in the way of my plans.	External

*Note*. The instruction is as follows: “The following statements may apply more or less to you. To what extent do you think each statement applies to you personally?”

## Method

### Samples

To investigate the psychometric properties of the English-language adaptation of the IE-4 scale and their comparability with those of the German-language source instrument, we assessed both versions in a Web-based survey conducted in the UK and Germany by the online access panel provider respondi AG using computer-assisted self-administered interviewing (CASI). Data collection took place in January 2018. For both nations, quota samples were drawn that represented the heterogeneity of the adult population in terms of age, gender, and educational attainment. Data from the last German Census (2011) were used as a reference (https://ergebnisse.zensus2011.de/?locale=en). To avoid bias introduced by a lack of reading/language proficiency, only native speakers of the respective languages were recruited. The purpose of the research (to investigate the quality of several questionnaires) was explained to the respondents, who were financially rewarded for their participation. Respondents consented to their participation in an anonymous online survey. Approval by an ethics committee was not necessary. In both nations, a subsample of the same participants who had participated in the main survey was reassessed after around 3 to 4 weeks (median time intervals: 28 days in the UK and 20 days in Germany).

Only respondents who completed the full questionnaire—that is, who did not abort the survey prematurely—were included in our analyses. The gross sample sizes were *N*_UK_ = 508 (retest: *N*_UK_ = 117) and *N*_DE_ = 513 (retest: *N*_DE_ = 125). We excluded 40 cases (7.9%) from the UK sample and 39 cases (7.6%) from the German sample based on three indicators: (a) ipsatized variance—that is, the within-person variance across items [45]—if the respondent fell within the lower 5% of the sample distribution of ipsatized variance; (b) the Mahalanobis distance of the respondent’s response vector from the average sample response vector [46] if the respondent fell within the upper 2.5% of the sample distribution of the Mahalanobis distance; and (c) implausibly short response times, namely, if the respondent took, on average, less than 1 s to respond to an item. Our intention in choosing relatively conservative cut-off values was to avoid excluding valid cases. All exclusion criteria were applied simultaneously—that is, any respondent who violated one or more of the three criteria was excluded from the analyses, and only those who met all three criteria were included. The final samples consisted of *N*_UK_ = 468 (retest: *N*_UK_ = 111) and *N*_DE_ = 474 (retest: *N*_DE_ = 117). Table 2 depicts in detail the sample characteristics and their distribution.

**Table 2:** Sample characteristics by nation.

	United Kingdom	Germany
*N*	468	474
Mean age in years (*SD*) [Range]	45.2 (14.5) [18–69]	44.0 (14.4) [18–69]
Proportion of women (%)	52.6	50.0
Educational level (%)		
Low: never went to school, Skills for Life/1–4 GCSEs A*–C or equivalent	34.8	33.5
Intermediate: 5 or more GCSEs A*–C/vocational GCSE/GNVQ intermediate or equivalent	32.1	33.8
High: 2 or more A-levels or equivalent	33.1	32.7

*Note*. The equivalent German educational levels were as follows (from low to high): no educational qualification/basic school-leaving qualification [German: *ohne Bildungsabschluss/Hauptschulabschluss*], intermediate school-leaving qualification [German: *Mittlere Reife*], entrance qualification for a university of applied sciences/general higher education entrance qualification [German: *Fachhochschulreife/Abitur*].

### Materials

The online surveys were conducted in German for the German sample and in English for the UK sample. Study questionnaires comprised the respective language version of IE-4, a set of questions on sociodemographic characteristics (i.e., gender, age, highest level of education, income, and employment status), and numerous measures to enable us to subsequently examine the relationship between scores on IE-4 and on scales measuring other constructs. Because IE-4 was part of a comprehensive multi-theme survey, our choice of correlates was driven by a combination of theoretical considerations and data availability.

On theoretical grounds, we selected, first, constructs that reflect general and domain-specific manifestations of psychological dispositions and resources: (a) the Big Five personality traits; (b) risk proneness; (c) impulsive behavior; (d) optimism; (e) general self-efficacy; (f) self-esteem. Second, we selected constructs that reflect social and political attitudes, values, and behaviors: (g) interpersonal trust; (h) internal and external political efficacy; and (i) justice sensitivity. Third, we selected a construct that reflects quality of life—namely, (j) life satisfaction.

As outlined in the theoretical background section, previous research has found that all these constructs consistently correlate with (internal or external) locus of control. Accordingly, we expected internal locus of control to correlate positively with the Big Five personality traits (the highest with Emotional Stability), risk proneness, impulsive behavior, optimism, general self-efficacy, self-esteem, interpersonal trust, internal political efficacy, and life satisfaction, as well as negatively with justice sensitivity. We further expected external locus of control to be positively related to life satisfaction, and justice sensitivity, as well as negatively to optimism, self-efficacy, self-esteem, and life satisfaction.

Fourth, we examined the susceptibility of IE-4 to two aspects of (k) socially desirable responding (exaggerating positive qualities and minimizing negative qualities) and, hence, a possible distortion of respondents’ answers. Therefore, the following short-scale measures were also administered as part of the survey, each in the respective language version:

- The well-established 15-item extra-short form of the Big Five Inventory–2 (BFI-2-XS; English-language version: [47]; German-language version: [48]) measures the Big Five dimensions Extraversion, Agreeableness, Conscientiousness, Emotional Stability, and Openness with three items per dimension. In the present study, internal consistency ranged between α = .44 (Openness) and α = .79 (Emotional Stability) in the UK sample, and between α = .37 (Agreeableness) and α = .68 (Emotional Stability) in the German sample. Rammstedt et al. [48] and Soto and John [47] showed evidence for factorial and construct validity.
- The 1-item Risk Proneness Short Scale (R-1) [49]/Kurzskala zur Erfassung der Risikobereitschaft [28] measures the willingness to take or tolerate risks. R-1 shows good test–retest stability (*r*_tt_ = .76 for the English-language version, *r*_tt_ = .83 for the German-language version) and evidence for construct validity [49].
- The 8-item Impulsive Behavior Short Scale–8 (I-8) [50]/Skala Impulsives Verhalten–8 [30] measures urgency, lack of premeditation, lack of perseverance, and sensation seeking with two items per subdimension. I-8 shows good internal consistency (ω = .65–.94 for the English-language version, ω = .65–.91 for the German-language version) and evidence for factorial and construct validity [50].
- The 2-item Optimism–Pessimism Short Scale–2 (SOP2) [51]/Skala Optimismus–Pessimismus–2 [52] measures dispositional optimism. SOP2 shows sufficient internal consistency (ω = .68 for the English-language version, ω = .77 for the German-language version) and evidence for factorial and construct validity [51].
- The 3-item General Self-Efficacy Short Scale–3 (GSE-3) [53]/Allgemeine Selbstwirksamkeit Kurzskala (ASKU) [23] measures the global confidence in one‘s own competence. GSE-3/ASKU shows good internal consistency (ω = .92 for the English-language version, ω = .86 for the German-language version) and evidence for factorial and construct validity [53].
- The 10-item Rosenberg Self-Esteem Scale (RSES; English-language version: [54]; German-language version: [55]) measures individual self-worth. In the present study, RSES showed good internal consistency (α = .90 in the UK sample, α = .89 in the German sample). Von Collani and Herzberg [55] showed evidence for factorial validity.
- The 3-item Interpersonal Trust Short Scale (KUSIV3) [56]/Kurzskala Interpersonelles Vertrauen [15] measures a person’s expectation that other persons and institutions can be relied on. KUSIV3 shows sufficient internal consistency (ω = .69 for the English-language version, ω = .75 for the German-language version) and evidence for factorial and construct validity [56].
- The 4-item Political Efficacy Short Scale (PESS) [57]/Political Efficacy Kurzskala (PEKS) [20] measures internal and external political efficacy with two items each. PESS/PEKS shows good internal consistency (ω = .84–.88 for the English-language version, ω = .86 for the German-language version) and evidence for factorial and construct validity [57].
- The 8-item Justice Sensitivity Short Scale–8 (JSS-8) [58]/Ungerechtigkeitssensibiliät-Skalen–8 (USS-8) [27] measures justice sensitivity from four perspectives (victim, observer, beneficiary, perpetrator) with two items each. JSS-8/USS-8 shows good internal consistency (ω = .76–.87 for the English-language version, ω = .73–.89 for the German-language version) and evidence for factorial and construct validity [58].
- The 1-item General Life Satisfaction Short Scale (L-1) [59]/Kurzskala zur Erfassung der Allgemeinen Lebenszufriedenheit [60] measures the cognitive-evaluative component of subjective well-being. L-1 shows good test–retest stability (*r*_tt_ = .82 for the English-language version, *r*_tt_ = .71 for the German-language version) and evidence for construct validity [59].
- The 6-item Social Desirability–Gamma Short Scale (KSE-G) [61]/Soziale Erwünschtheit–Gamma [62] measures two aspects of socially desirable responding—exaggerating positive qualities and minimizing negative qualities—with three items per subdimension. KSE-G shows sufficient internal consistency (ω = .67–.79 for the English-language version, ω = .69–.70 for the German-language version) and evidence for factorial and construct validity [61].

To assess income, respondents were asked to allocate their net income to one of 17 categories ranging from 1 (*less than £200* [DE: *300 euros*]) to 17 (*£10*,*000* [DE: *10*,*000 euros*] *and more*). An 18th category (*no personal income*) was provided for those who had no income. None of the participants chose that category. Before computing the correlations, we recoded the negatively worded items of all short scales (for both language versions), the subdimension “minimizing negative qualities” of socially desirable responding (for both language versions), and the self-esteem scale (UK only), so that high values always represented high levels of the respective traits. Because the Big Five dimension Emotional Stability is negatively worded in relation to the construct Negative Emotionality in the BFI-2-XS, we recoded the respective items so that high values represented the positive pole of this dimension—that is, Emotional Stability. In addition, we recoded the employment status variable and tested two contrasts: (a) unemployed (out of work and looking for work/out of work but not currently looking for work) versus (self-)employed, and (b) retired/doing housework versus (self-)employed. We did not make further contrasts, and regarded all other employment status categories (i.e., pupil/student, apprentice/intern) as missing values because the sample sizes of these categories were too small.

## Results

To empirically examine the English-language adaptation of IE-4, and to investigate its comparability with the German-language source version, we analyzed the psychometric properties objectivity, reliability, and validity in both language versions. Moreover, we assessed measurement invariance across both nations. The statistical analyses were run with R (for the R packages used, see corresponding subsections below). The code can be found in the S3 Appendix.

### Descriptive statistics and reference ranges

In the first step, we analyzed the descriptive statistics and reference ranges for the German- and English-language versions of IE-4 separately. Table 3 shows the means, standard deviations, skewness, and kurtosis for the four items as well as for the two mean subscale scores, separately for the UK and German samples. All descriptive statistics were comparable across the two language versions. They showed that internal locus of control was slightly right-skewed, whereas external locus of control was slightly left-skewed. The inter-scale correlations (*r* = .12 in the UK and *r* = −.29 in the German sample) and the inter-item correlations (see Table 3) revealed that the two subscales were more independent of each other in the UK than in Germany. Interestingly, internal and external locus of control were positively correlated in the UK, and negatively correlated in Germany.

**Table 3:** Descriptive statistics and inter-item correlations by nation for the IE-4 items.

	*M*		*SD*		Skewness		Kurtosis		Item 2		Item 3		Item 4	
	UK	DE	UK	DE	UK	DE	UK	DE	UK	DE	UK	DE	UK	DE
Item 1	3.20	4.14	1.40	0.79	−0.21	−0.80	−1.22	0.69	.43	.51	−.00	−.26	.16	−.23
Item 2	3.60	3.91	1.15	0.85	−0.55	−0.67	−0.51	0.42			.07	−.18	.14	−.17
Item 3	2.33	2.24	1.17	1.03	0.69	0.71	−0.39	0.07					.46	.42
Item 4	2.60	2.80	1.13	1.06	0.32	0.26	−0.66	−0.49						

*Note*. The rating scale ranged from 1 (*low*) to 5 (*high*). UK = United Kingdom (*N =* 468); DE = Germany (*N =* 474).

Table 4 provides the reference ranges in terms of the means, standard deviations, skewness, and kurtosis of the IE-4 scale scores for the total population as well as separately for gender and age groups in both nations.

**Table 4:** Reference ranges of the IE-4 scale scores by nation for the total population and separately for gender and age groups.

	*M*		*SD*		Skewness		Kurtosis	
	UK	DE	UK	DE	UK	DE	UK	DE
Internal locus of control	3.40	4.02	1.08	0.71	−0.29	−0.84	−0.70	0.97
External locus of control	2.46	2.52	0.98	0.88	0.63	0.61	−0.06	0.22
Internal locus of control								
Male [*n*_UK_ = 222; *n*_DE_ = 237]	3.37	3.99	1.08	0.71	−0.25	−0.89	−0.74	1.38
Female [*n*_UK_ = 246; *n*_DE_ = 237]	3.43	4.06	1.08	0.71	−0.33	−0.79	−0.69	0.50
External locus of control								
Male [*n*_UK_ = 222; *n*_DE_ = 237]	2.49	2.48	1.06	0.88	0.56	0.70	−0.45	0.51
Female [*n*_UK_ = 246; *n*_DE_ = 237]	2.43	2.55	0.91	0.88	0.68	0.51	0.35	−0.06
Internal locus of control								
Age 18−29 [*n*_UK_ = 104; *n*_DE_ = 105]	3.38	4.13	1.05	0.69	−0.20	−0.78	−0.49	0.18
Age 30−49 [*n*_UK_ = 180; *n*_DE_ = 191]	3.38	4.00	1.09	0.76	−0.34	−0.94	−0.80	1.41
Age 50−69 [*n*_UK_ = 184; *n*_DE_ = 178]	3.43	3.98	1.09	0.66	−0.29	−0.70	−0.77	0.42
External locus of control								
Age 18−29 [*n*_UK_ = 104; *n*_DE_ = 105]	2.41	2.57	1.05	0.93	0.91	0.56	0.33	−0.12
Age 30−49 [*n*_UK_ = 180; *n*_DE_ = 191]	2.69	2.60	1.01	0.89	0.31	0.51	−0.62	0.04
Age 50−69 [*n*_UK_ = 184; *n*_DE_ = 178]	2.26	2.40	0.86	0.82	0.70	0.70	0.42	0.65

*Note*. UK = United Kingdom (*N =* 468); DE = Germany (*N =* 474).

### Objectivity

A scale can be regarded as objective when it is independent of (a) the administrator (objectivity of application) and (b) the evaluator of the instrument (objectivity of evaluation), and when (c) unambiguous and user-independent rules are provided (objectivity of interpretation). The standardized questionnaire format and written instructions, the fixed scoring rules and labeled response categories, and the reference ranges ensured the objectivity of the application, evaluation, and interpretation of IE-4.

### Reliability

As estimates for the internal reliability of IE-4, we estimated McDonald’s omega (ω) [63, 64] based on the CFA model, using the R package “semTools” [65]. In addition, we computed the test–retest stability of the observed scale scores, *r*_tt_, over a period of about 28 days (*Mdn*) in the UK (*N*_UK_ = 111) and 20 days (*Mdn*) in Germany (*N*_DE_ = 117), respectively. Our reasoning was that this time span of 3 to 4 weeks was long enough to allow for meaningful test–retest stability estimates and short enough to preclude the occurrence of pronounced and systematic change in the true scores of internal and external locus of control. Because the test–retest stability is sensitive not only to measurement error but also to state fluctuations in dispositional internal and external locus of control [66], the resulting reliability coefficient is best understood as a lower-bound estimate.

The reliability estimates for IE-4 are reported in Table 5. In detail, IE-4 proved to be comparably reliable in both samples. In such cases, test–retest correlations are recommended for a comparison of the reliability of scale scores. However, especially given the small number of items, not only test–retest estimates (r_*tt*_) but also internal consistency estimates (ω) are satisfactory and sufficient for research purposes [67, 68] because to capture a construct more broadly and not too homogeneously with only two items per dimension always comes at the expense of internal consistency. However, if latent-variable models are used that correct for unreliability, the reliability of the observed scale scores is not important.

**Table 5:** Reliability estimates of IE-4.

	ω		*r*_tt_ [95% CI]	
	UK	DE	UK	DE
Internal locus of control	.59	.67	.71 [.60, .79]	.67 [.55, .76]
External locus of control	.63	.59	.64 [.51, .74]	.61 [.48, .71]

*Note*. UK = United Kingdom (*N =* 468; retest: *N* = 111); DE = Germany (*N =* 474; retest: *N* = 117); CI = confidence interval. The time interval between test and retest ranged between 15 and 31 days (*Mdn*_UK_ = 28 days; *Mdn*_DE_ = 20 days).

### Validity

Content-related validity evidence was provided by Kovaleva [40] and Kovaleva et al. [4] during the development of the original, German-language, scale. In addition, we investigated two types of validity evidence—namely, evidence based on the internal structure of the scale and evidence based on the relationship between scores on the IE-4 scale and on scales measuring other variables.

#### Validity evidence based on the internal structure of IE-4

We investigated the factorial structure of IE-4 separately in the UK and Germany by means of CFA with the R package “lavaan” [69] using a two-dimensional measurement model developed for the German-language scale by Kovaleva et al. [4] with two latent factors capturing internal and external locus of control, respectively. We identified the models by fixing the first intercept of each latent factor to 0 and the first factor loading to 1. For all models, we used robust maximum likelihood (MLR) estimation.

We first fit a just-identified congeneric model. Identification via latent covariances may result in a relatively unstable model, which was evident by the negative residual variance of the fourth item in the UK. Therefore, we restricted the variance to be higher than 0. No fit indices are available for this model because it has no degrees of freedom. That is why we next estimated an essentially tau-equivalent model with unit factor loadings (i.e., setting all factor loadings to 1). The fit indices refer to the commonly used MLR-scaled comparative fit index (CFI) and the root mean square error of approximation (RMSEA), which are functions of the MLR-adjusted chi-square statistic. According to the rules of thumb for a good model fit proposed by Hu and Bentler [70], the model fit was very good for Germany, and not quite as good but still acceptable for the UK, apart from a slightly too high RMSEA (but see Browne & Cudeck [71], according to whom an RMSEA of this size would still be acceptable): UK—χ^2^(3) = 10.571, *p* = .014, CFI = .959, RMSEA = .073, SRMR (standardized root mean square residual) = .033, BIC (Bayesian information criterion) = 5,870.508; DE—χ^2^(3) = 1.788, *p* = .618, CFI = 1.000, RMSEA = .000, SRMR = .017, BIC = 4,863.359. Because the commonly used MLR-scaled CFI and RMSEA lead to biased population values, R/lavaan additionally provides so-called robust CFI and robust RMSEA values that prevent biased fit indices, [72, 73]: UK—robust CFI = .964, robust RMSEA = .075; DE—robust CFI = 1.000, robust RMSEA = .000.

**Fig 1:**
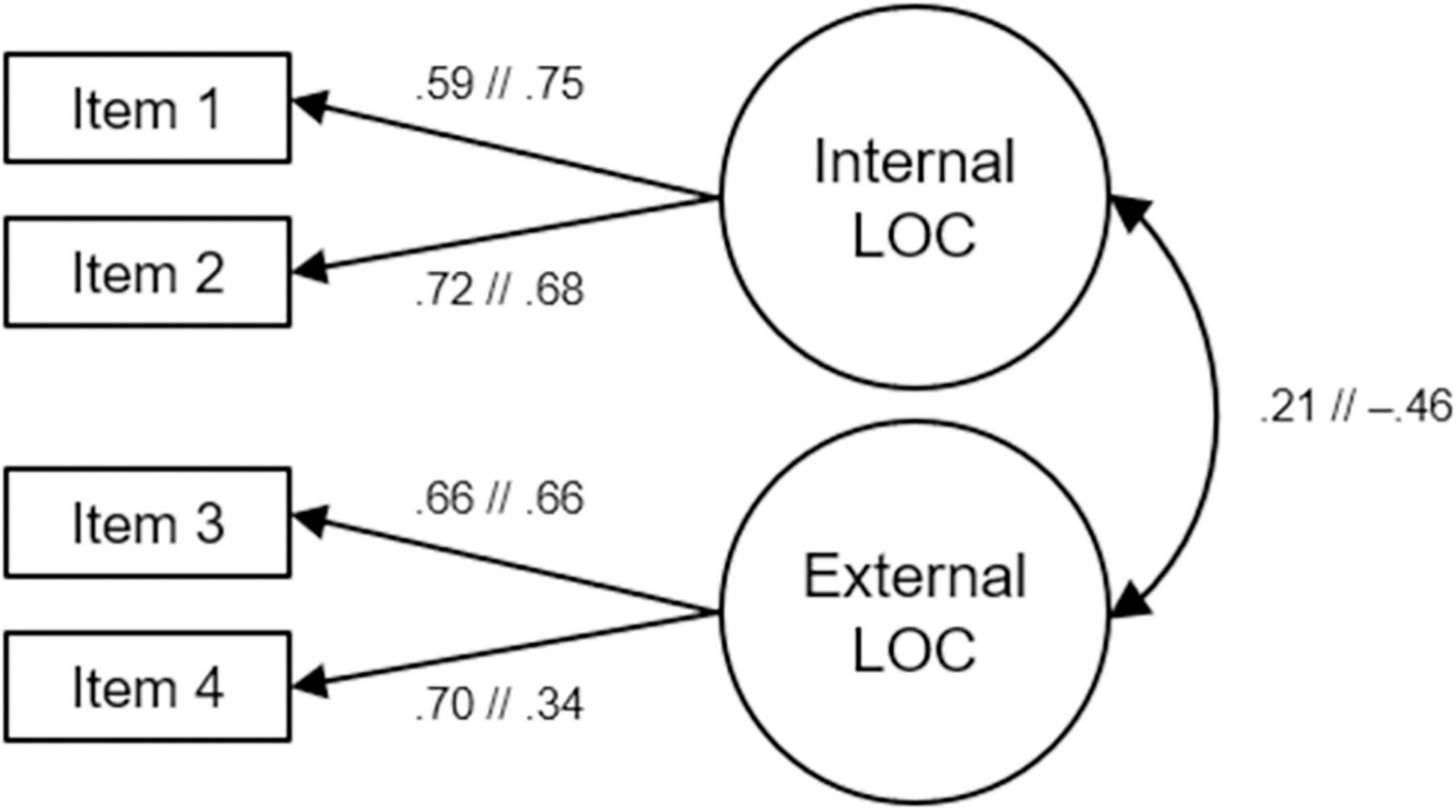
Two-dimensional measurement model of IE-4 with Standardized coefficients and equalized factor loadings. *Note*. LOC = locus of control. The coefficients of the German sample are presented after the double slash. Item error terms have been omitted for clarity. *N*_UK_ = 468; *N*_DE_ = 474.

The items’ factor loadings are depicted in Fig 1. The factor loadings of three of the four items (Items 1–3) were very similar in both nations. By contrast, the factor loading of Item 4 in the German sample was only half as high as in the UK sample. Moreover, the correlation between internal and external locus of control was positive in the UK sample (*r* = .21), but it was negative and more than twice as large in the German sample (*r* = –.46). This large negative correlation is consistent with the results of Kovaleva [40] and Kovaleva et al. [4], who found an even larger negative association (*r* = –.62) between internal and external locus of control in the original scale-development process.

#### Validity evidence based on the relationship between scores on IE-4 and on scales measuring other variables

Evidence based on the relationship between scores on IE-4 and on scales measuring other variables was gained from manifest indicators (scale scores). The correlation coefficients are depicted in Table 6. Their interpretation is based on effect size guidelines proposed by Gignac and Szodorai [74]: relatively small effects (*r* ≥ .10), typical (medium) effects (*r* ≥ .20), and relatively large effects (*r* ≥ .30). According to these authors, a correlation of .19 corresponds to the 50th percentile of a meta-analytical distribution of correlations in individual differences research. Therefore, in Table 6, medium to large effects are highlighted. We did not test hypotheses or computed a null hypothesis significance test (NHST) but examined the nomological network exploratively. Therefore, we did not consider *p*-values but focused on effect sizes only. In order to investigate validity evidence based on relations with other constructs, we correlated scores on IE-4 with scores on the scales outlined in the Materials section. Table 6 displays all single correlations. For the analysis of correlations between IE-4 and three scales/items with some missing values (see the notes of Table 6), we used pairwise deletion. In the following, we select and describe in detail only a few correlations that appeared to be the most noteworthy.

**Table 6:** Correlations of IE-4 with validation measures and sociodemographic characteristics, by nation.

	Internal locus of control				External locus of control			
	UK		DE		UK		DE	
	*r*	95% CI	*r*	95% CI	*r*	95% CI	*r*	95% CI
Big Five								
Extraversion	**.25**	[.16, .33]	**.25**	[.17, .33]	–.01	[–.10, .09]	–.13	[–.22,–.04]
Agreeableness	.06	[–.03, .15]	**.20**	[.12, .29]	–.02	[–.11, .07]	–.13	[–.22,–.04]
Conscientiousness	.16	[.07, .25]	**.26**	[.17, .34]	**–.24**	[–.32,–.15]	–.15	[–.24,–.06]
Emotional Stability	.19	[.10, .27]	**.30**	[.22, .38]	**–.20**	[–.28,–.11]	**–.42**	[–.49,–.35]
Openness	**.21**	[.12, .30]	.16	[.07, .24]	.00	[–.09, .09]	–.04	[–.13, .05]
Risk proneness	**.23**	[.14, .31]	.19	[.10, .27]	.16	[.07, .25]	–.00	[–.09, .09]
Impulsive behavior								
Urgency	.12	[.03, .21]	–.03	[–.12, .06]	**.39**	[.32, .46]	**.24**	[.15, .32]
Premeditation	**.25**	[.16, .33]	.18	[.09, .26]	.15	[.06, .24]	–.07	[–.16, .02]
Perseverance	**.36**	[.28, .43]	**.43**	[.35, .50]	.16	[.07, .25]	–.15	[–.24,–.06]
Sensation seeking	**.31**	[.23, .39]	**.26**	[.18, .34]	**.24**	[.16, .33]	.05	[–.04, .14]
Optimism	**.27**	[.18, .35]	**.28**	[.20, .36]	–.18	[–.27,–.09]	**–.39**	[–.47,–.31]
General self-efficacy	**.38**	[.30, .45]	**.51**	[.44, .57]	–.01	[–.10, .08]	**–.22**	[–.31,–.14]
Self-esteem	**.27**	[.18, .35]	**.40**	[.32, .47]	**–.27**	[–.35,–.19]	**–.48**	[–.54,–.40]
Interpersonal trust	.16	[.08, .25]	.16	[.07, .24]	–.06	[–.15, .04]	**–.22**	[–.30,–.13]
Political efficacy								
Internal	**.25**	[.16, .33]	.17	[.08, .25]	.16	[.07, .25]	–.07	[–.16, .02]
External	**.22**	[.14, .31]	.01	[–.08, .10]	**.33**	[.25, .41]	.09	[.00, .18]
Justice sensitivity								
Victim	–.05	[–.14, .04]	–.06	[–.15, .03]	**.33**	[.25, .41]	**.25**	[.17, .33]
Observer	.00	[–.09, .09]	.03	[–.06, .12]	**.21**	[.12, .29]	.11	[.02, .20]
Beneficiary	.02	[–.07, .11]	–.03	[–.12, .06]	**.26**	[.17, .34]	.11	[.02, .19]
Perpetrator	.06	[–.03, .15]	.05	[–.04, .14]	.08	[–.02, .17]	–.05	[–.14, .04]
Life satisfaction	**.25**	[.17, .34]	**.35**	[.27, .43]	–.12	[–.21,–.03	**–.43**	[–.50,–.35]
Social desirability								
PQ+	**.27**	[.18, .35]	**.29**	[.21, .37]	.05	[–.04, .14]	–.15	[–.23,–.06]
NQ–	–.11	[–.20,–.02]	.09	[–.00, .18]	**–.34**	[–.42,–.26]	–.16	[–.24,–.07]
Sociodemographic characteristics								
Employed (= reference category)								
Unemployed	–.09	[–.19, .02]	**–.21**	[–.31,–.10]	.05	[–.06, .15]	.10	[–.02, .21]
Retired/homemaker	.11	[.01, .21]	–.09	[–.19, .01]	–.09	[–.19, .01]	.01	[–.09, .11]
Income	.16	[.07, .25]	**.25**	[.16, .33]	–.01	[–.11, .08]	–.19	[–.27,–.09]
Educational level	.09	[.00, .18]	.03	[–.06, .12]	–.03	[–.12, .06]	–.11	[–.20,–.02]
Age	.04	[–.06, .13]	–.05	[–.14, .04]	–.14	[–.23,–.05]	–.09	[–.18,–.00]
Gender	.03	[–.06, .12]	.05	[–.04, .14]	–.03	[–.12, .06]	.04	[–.05, .13]

*Note*. UK = United Kingdom (*N =* 468, *N*_Employment status_ = 450, *N*_Income_ = 431); DE = Germany (*N =* 474, *N*_Self-esteem_ = 473, *N*_Employment status_ = 462, *N*_Income_ = 449); CI = confidence interval; PQ+ = exaggerating positive qualities; NQ– = minimizing negative qualities. Gender: 1 = male, 2 = female. Coefficients with *r* ≥ |.20| are in bold type.

In both nations, internal locus of control showed the largest positive associations with general self-efficacy and the impulsive behavior subscale perseverance. This is in line with previous findings that individuals who believe that an event is dependent on their own behavior/personality also tend to have greater confidence in their own competencies (i.e., general self-efficacy) [4, 24] and a greater ability to keep focused on (boring/difficult) tasks, even in the presence of distractions [75] (i.e., perseverance [4, 47]).

Previous research has suggested positive correlations between internal locus of control and self-esteem [25], life satisfaction [4, 22], optimism [4, 32], and Emotional Stability [4, 76], and negative correlations between external locus of control and these constructs [4, 22, 25, 32, 76]. We could replicate these patterns for both nations with small-to-large-sized effects. Individuals with higher self-esteem, higher life satisfaction, and higher optimism had a higher propensity to believe that an event was dependent on their own behavior/personality rather than a result of chance, or under the control of others.

In addition, we found that both internally and externally controlled persons were susceptible to socially desirable responding. Internal locus of control was associated with exaggerating positive qualities, whereas external locus of control was associated with minimizing negative qualities. In other words, individuals who tended to exaggerate positive qualities also had a tendency to believe that an event was dependent on their own behavior/personality. By contrast, individuals who tended to minimize negative qualities had a tendency to believe that an event was the result of chance, or was under the control of others.

We calculated correlations between IE-4 and relevant sociodemographic characteristics—namely, employment status, income, educational level, age, and gender. We found only a medium-sized positive correlation between internal locus of control and income in Germany, and a small-sized negative correlation between external locus of control and income in Germany. The latter findings are in line with evidence from Kovaleva et al. [4] indicating that internal locus of control increases, and external locus of control decreases with increasing income.

Despite some differences in the patterns of correlations between the UK and Germany, the overlapping confidence intervals suggest that many of these differences were statistically non-significant. Overall, the nomological networks were fairly similar, albeit not identical, across nations. The profile similarities (i.e., correlations between the vector of nomological correlations between the UK and Germany) were *r* = .59, 95% CI [.29, .79] for internal locus of control and *r* = .56, 95% CI [.25, .77] for external locus of control.

### Cross-national comparability

We assessed the comparability of IE-4 across the UK and Germany via measurement invariance tests with multiple-group confirmatory factor analyses (MG-CFA) [77, 78]. The measurement invariance tests were based on the essentially tau-equivalent two-dimensional model with equal loadings using MLR estimation. Therefore, the configural model and the metric model are equivalent. We identified the mean structure of the model by fixing the first intercept to 0, and we identified the covariance structure by fixing the first loading to 1. In order to determine the level of measurement invariance, we used the cut-off values recommended by Chen [79]. According to these benchmarks, metric invariance must be rejected when the χ^2^ difference test is significant and/or ΔCFI ≤ −.010 either in combination with ΔRMSEA ≥ .015 or ΔSRMR ≥ .030; scalar and full uniqueness invariance must be rejected when the χ^2^ difference test is significant and/or ΔCFI ≤ −.010 either in combination with ΔRMSEA ≥ .015 or ΔSRMR ≥ .010.

Because the metric model showed a good fit—χ^2^(6) = 11.382, *p* = .077, CFI = .986 (robust CFI = .987), RMSEA = .044 (robust RMSEA = .047), SRMR = .025, BIC = 10,749.117—metric invariance can be accepted, implying the comparability of correlations based on the latent factors between both nations. When comparing the scalar model—χ^2^(6) = 86.554, *p* < .000, CFI = .793 (robust CFI = .820), RMSEA = .144 (robust RMSEA = .153), SRMR = .065, BIC = 10,819.123—with the metric model, the significant χ^2^ difference and the MLR-scaled CFI indicated that scalar invariance of IE-4 did not hold across the UK and Germany: Δχ^2^(2) = 84.548, *p* < .001, ΔCFI = –.193 (Δ robust CFI = –.167), ΔRMSEA = .010 (Δ robust RMSEA = .106), ΔSRMR = .004, ΔBIC = –70.006.

## Discussion and conclusion

The aim of the present study was, first, to empirically assess the psychometric properties of the Internal–External Locus of Control Short Scale–4 (IE-4), the English-language adaptation of the German-language source version Internale-Externale-Kontrollüberzeugung-4, developed by Kovaleva et al. [4]. Second, we aimed to examine the cross-national comparability of the two scales across the UK and Germany via measurement invariance tests. Our results were based on two comprehensive quota samples representing the heterogeneity of the adult populations in the UK and Germany. The results show, first, that the English-language version of IE-4 is a reliable and valid ultra-short instrument to measure internal and external locus of control. Second, they show that the psychometric properties of the two language versions are largely comparable in the case of reliability estimates, the factorial structure, and some correlates with external variables. Third, the results of measurement invariance testing suggest metric invariance of the scale, thereby implying the comparability of variances and covariances based on the latent factors across the UK and Germany.

The non-achievement of scalar invariance indicates that the latent means between the UK and Germany are not comparable without systematic bias, implying that the two countries do not have the same point of origin. A possible reasons for this could be, for example, that the two nations used different frames of reference when assessing their locus of control beliefs [80]. To account for this variability, we conducted a multiple indicators multiple causes (MIMIC) model by regressing the non-invariant item (Item 2) and the latent variables on the country variable in the scalar model. The results showed that the country significantly predicted both the non-invariant item and the corresponding latent variable of internal locus of control (but not the latent variable of external locus of control), indicating that Item 2 functions differently across countries and is thus non-invariant. To be precise, the UK nation was associated with almost 1 scale point lower internal locus of control compared to the German nation. We recommend users who want to use the scale for international comparisons to recheck measurement invariance and⸺if scalar invariance does not hold in their sample⸺not to compare latent means.

We could replicate the two-dimensional structure of internal and external locus of control in both language versions. As expected, in most cases, these two dimensions were differentially associated with a total of 11 other psychological constructs, which we used to embed the scale in a nomological network. In other words, either the direction of the effects (i.e., the algebraic signs) or the effect sizes differed, or both. Taken together, we could, for the most part, support and expand the findings of the original validation study of the German-language source version [4] with respect to these correlations.

In addition, there were also some discernible differences between the two nations, both in terms of the direction of the effects and of their strength. In the UK, we found, for example, a small positive effect between external locus of control and scores on the impulsive behavior subscale perseverance, whereas in Germany we found a small negative effect between the two variables. Moreover, there were medium-to-large-sized positive associations between both locus of control dimensions and external political efficacy in the UK, whereas there were zero correlations in Germany. By contrast, we found a similar pattern for the relation between internal locus of control and Agreeableness, but in this case with a medium-sized positive effect in Germany and a zero correlation in the UK.

The size of the factor loading of Item 4 was also different between samples, and was twice as large in the UK as in the German sample. Furthermore, the correlation between internal and external locus of control also differed across nations: whereas in the UK, there was just a medium-sized positive association, the association in Germany was negative and twice as large as that in the UK. All these differences may be due to national specificities, such as differing socialization. However, this is purely speculative; further research is needed to explore these cross-national differences in depth. Moreover, future studies could also examine correlations between IE-4 and more fine-grained subdimensions/facets of each construct in order to further elaborate the nomological network of IE-4. In doing so, a network analysis could be performed to show the graphical representation of the correlation matrix.

Despite the benefits of our study, its scope was limited in three ways. First, both samples were restricted to participants in a Web-based survey (CASI); second, the English-language sample was restricted to the population of the UK. Consequently, we cannot generalize our findings to the whole population—including, for example, persons who are not computer literate—or to other English-speaking populations, for example, in the United States. Although there is no reason to expect major differences between survey modes or English-speaking nations, future research might address these issues. Third, due to survey time constraints (IE-4 was administered as part of a comprehensive online survey for the validation of various short scales), we could not include alternative measures of internal and external locus of control in our study. However, previous research has reported that the two dimensions of the German-language source scale of IE-4 (i.e., internal and external locus of control) correlated highly with the corresponding dimensions of another locus of control scale, KMKB [39], which has similar good psychometric properties as IE-4 (*r* = .92–.99) [4, 40], indicating evidence for convergent validity.

To conclude, the results of the present validation study show for the first time the utility and psychometric properties of the English-language adaptation of the ultra-short IE-4 scale and the comparability of its psychometric properties with those of the German-language source version. Researchers in English-speaking nations now have the possibility of measuring internal and external locus of control in an economical and time-efficient way in assessment settings with limited resources, such as large-scale surveys in the social sciences. We recommend applying IE-4 only in self-report surveys for research purposes in measurement settings with severe time limitations, and not for individual diagnostics.

## Supporting information

S1 Appendix Answer sheet (German-language version). IE-4.
(PDF)

S2 Appendix Answer sheet (English-language version). IE-4.
(PDF)

S3 Appendix R code for analysis. (PDF)
