# Supplementary material for: The Internal–External Locus of Control Short Scale–4 (IE-4): A comprehensive validation of the English-language adaptation
Source: PLoS One. 2022 Jul 11;17(7):e0271289. doi: 10.1371/journal.pone.0271289 (PMC9273068; doi:10.1371/journal.pone.0271289)
Supplement: S1 Appendix — IE-4. (PDF) [file pone.0271289.s001.pdf]

S1 Appendix: Answer Sheet (German-Language Version)

Internale-Externale-Kontrollüberzeugung-4 (IE-4)

Die folgenden Aussagen können mehr oder weniger auf Sie zutreffen. Bitte geben Sie bei jeder Aussage an, inwieweit diese auf Sie persönlich zutrifft.

		trifft gar nicht zu	trifft wenig zu	trifft etwas zu	trifft ziemlich zu	trifft voll und ganz zu
1.	Ich habe mein Leben selbst in der Hand.	□	□	□	□	□
2.	Wenn ich mich anstrenge, werde ich auch Erfolg haben.	□	□	□	□	□
3.	Egal ob privat oder im Beruf: Mein Leben wird zum großen Teil von anderen bestimmt.	□	□	□	□	□
4.	Meine Pläne werden oft vom Schicksal durchkreuzt.	□	□	□	□	□

Source: Kovaleva et al. [4]
