# Supplementary material for: The Internal–External Locus of Control Short Scale–4 (IE-4): A comprehensive validation of the English-language adaptation
Source: PLoS One. 2022 Jul 11;17(7):e0271289. doi: 10.1371/journal.pone.0271289 (PMC9273068; doi:10.1371/journal.pone.0271289)
Supplement: S2 Appendix — IE-4. (PDF) [file pone.0271289.s002.pdf]

S2 Appendix: Answer Sheet (English-Language Version)

Internal–External Locus of Control Short Scale–4 (IE-4)

The following statements may apply more or less to you. To what extent do you think each statement applies to you personally?

	does not apply at all	applies a bit	applies somewhat	applies mostly	applies completely
1. I'm my own boss.	☐	☐	☐	☐	☐
2. If I work hard, I will succeed.	☐	☐	☐	☐	☐
3. Whether at work or in my private life: What I do is mainly determined by others.	☐	☐	☐	☐	☐
4. Fate often gets in the way of my plans.	☐	☐	☐	☐	☐
