# Supplementary material for: The Internal–External Locus of Control Short Scale–4 (IE-4): A comprehensive validation of the English-language adaptation
Source: PLoS One. 2022 Jul 11;17(7):e0271289. doi: 10.1371/journal.pone.0271289 (PMC9273068; doi:10.1371/journal.pone.0271289)
Supplement: S3 Appendix — (PDF) [file pone.0271289.s003.pdf]

S3 Appendix: R Code for Analysis

```
##########
#Analysis
##########

#Clear workspace (run if desired)
rm(list = ls())

# #List of project directories
# dirs <- list(
#   data = "...",
#   analysis = "...")

#Load required packages
if (!require(psych))  { install.packages("psych") } ; library(psych)
if (!require(lavaan)) { install.packages("lavaan") } ; library(lavaan)
if (!require(car)) { install.packages("car") } ; library(car)
if (!require(semTools)) { install.packages("semTools") } ; library(semTools)

#Load dataset
load(paste0(dirs$data, "IE-4.Rda"))

#Split dataset between countries
IE4_UK <- subset(IE4, subset = (COUN == "2"))
IE4_D <- subset(IE4, subset = (COUN == "1"))

#########################################################################

###########################
#Step 1: Measurement model
###########################

# #Tau-congeneric model
# IE4_MM_con1 <- 'LV_ILOC =~ ILOC1 + ILOC2
#                LV_ELOC =~ ELOC1 + ELOC2
#                ILOC1+ELOC1 ~ 0*1
#                LV_ILOC+LV_ELOC ~ NA*1'
#
# IE4_MM.fit <- sem(IE4_MM_con1, data = IE4_UK, estimator = "mlr", missing = "fiml", 
std.lv = FALSE)
# summary(IE4_MM.fit, standardized = TRUE, fit.measures = TRUE)
# #--> the residual variance of ELOC2 is negative in the UK
#
```

```
# IE4_MM.fit <- sem(IE4_MM_con1, data = IE4_D, estimator = "mlr", missing = "fiml",
std.lv = FALSE)
# summary(IE4_MM.fit, standardized = TRUE, fit.measures = TRUE)

#Essentially tau-equivalent model
IE4_MM_tau <- 'LV_ILOC =~ c(a1)*ILOC1 + c(a1)*ILOC2
        LV_ELOC =~ c(a2)*ELOC1 + c(a2)*ELOC2
        ILOC1+ELOC1 ~ 0*1
        LV_ILOC+LV_ELOC ~ NA*1'

IE4_MM.fit <- sem(IE4_MM_tau, data = IE4_UK, estimator = "mlr", missing = "fiml", std.lv
= FALSE)
summary(IE4_MM.fit, standardized = TRUE, fit.measures = TRUE)

IE4_MM.fit <- sem(IE4_MM_tau, data = IE4_D, estimator = "mlr", missing = "fiml", std.lv =
FALSE)
summary(IE4_MM.fit, standardized = TRUE, fit.measures = TRUE)

##################################################################################

##################################
#Step 2: Descriptive Statistics
##################################

##UK

describe((IE4_UK$ILOC1+IE4_UK$ILOC2)/2)
describe(IE4_UK$ILOC1)
describe(IE4_UK$ILOC2)

describe((IE4_UK$ELOC1+IE4_UK$ELOC2)/2)
describe(IE4_UK$ELOC1)
describe(IE4_UK$ELOC2)

cor.test((IE4_UK$ILOC1+IE4_UK$ILOC2), (IE4_UK$ELOC1+IE4_UK$ELOC2), use =
"pairwise.complete.obs")
cor.test(IE4_UK$ILOC1, IE4_UK$ILOC2, use = "pairwise.complete.obs")
cor.test(IE4_UK$ILOC1, IE4_UK$ELOC1, use = "pairwise.complete.obs")
cor.test(IE4_UK$ILOC1, IE4_UK$ELOC2, use = "pairwise.complete.obs")
cor.test(IE4_UK$ILOC2, IE4_UK$ELOC1, use = "pairwise.complete.obs")
cor.test(IE4_UK$ILOC2, IE4_UK$ELOC2, use = "pairwise.complete.obs")
cor.test(IE4_UK$ELOC1, IE4_UK$ELOC2, use = "pairwise.complete.obs")
```

```
##############################################################################

##Germany

describe((IE4_D$ILOC1+IE4_D$ILOC2)/2)
describe(IE4_D$ILOC1)
describe(IE4_D$ILOC2)

describe((IE4_D$ELOC1+IE4_D$ELOC2)/2)
describe(IE4_D$ELOC1)
describe(IE4_D$ELOC2)

cor.test((IE4_D$ILOC1+IE4_D$ILOC2), (IE4_D$ELOC1+IE4_D$ELOC2), use = 
"pairwise.complete.obs")
cor.test(IE4_D$ILOC1, IE4_D$ILOC2, use = "pairwise.complete.obs")
cor.test(IE4_D$ILOC1, IE4_D$ELOC1, use = "pairwise.complete.obs")
cor.test(IE4_D$ILOC1, IE4_D$ELOC2, use = "pairwise.complete.obs")
cor.test(IE4_D$ILOC2, IE4_D$ELOC1, use = "pairwise.complete.obs")
cor.test(IE4_D$ILOC2, IE4_D$ELOC2, use = "pairwise.complete.obs")
cor.test(IE4_D$ELOC1, IE4_D$ELOC2, use = "pairwise.complete.obs")

##############################################################################

######################
#Step 3: Reliability
######################

##UK

#McDonald's omega
IE4_MM_tau <- 'LV_ILOC =~ c(a1)*ILOC1 + c(a1)*ILOC2
        LV_ELOC =~ c(a2)*ELOC1 + c(a2)*ELOC2
        ILOC1+ELOC1 ~ 0*1
        LV_ILOC+LV_ELOC ~ NA*1'
IE4_MM.fit_UK <- sem(IE4_MM_tau, data = IE4_UK, estimator = "mlr", missing = "fiml", 
std.lv = FALSE)
semTools::reliability(IE4_MM.fit_UK)

#Retest reliability
IE4_UK$ILOC <- IE4_UK$ILOC1+IE4_UK$ILOC2
IE4_UK$ELOC <- IE4_UK$ELOC1+IE4_UK$ELOC2
IE4_UK$ILOCrt <- IE4_UK$ILOC1rt+IE4_UK$ILOC2rt
IE4_UK$ELOCrt <- IE4_UK$ELOC1rt+IE4_UK$ELOC2rt
```

```
cor.test(IE4_UK$ILOC, IE4_UK$ILOCrt, use = "pairwise.complete.obs")
cor.test(IE4_UK$ELOC, IE4_UK$ELOCrt, use = "pairwise.complete.obs")

#Cronbach's alpha (BFI-2-XS and RSES)
alpha(subset(IE4_UK, select = c(EXTR1R, EXTR2, EXTR3)))
alpha(subset(IE4_UK, select = c(AGRE1, AGRE2R, AGRE3)))
alpha(subset(IE4_UK, select = c(CONS1R, CONS2R, CONS3)))
alpha(subset(IE4_UK, select = c(NEGA1, NEGA2, NEGA3R)))
alpha(subset(IE4_UK, select = c(OPEN1, OPEN2R, OPEN3)))

alpha(subset(IE4_UK,
        select = c(RSES1, RSES2R, RSES3, RSES4, RSES5R, RSES6R, RSES7, RSES8R,
RSES9R, RSES10)))

###########################################################################

##Germany

#McDonald's omega
IE4_MM_tau <- 'LV_ILOC =~ c(a1)*ILOC1 + c(a1)*ILOC2
           LV_ELOC =~ c(a2)*ELOC1 + c(a2)*ELOC2
           ILOC1+ELOC1 ~ 0*1
           LV_ILOC+LV_ELOC ~ NA*1'
IE4_MM.fit_D <- sem(IE4_MM_tau, data = IE4_D, estimator = "mlr", missing = "fiml",
std.lv = FALSE)
semTools::reliability(IE4_MM.fit_D)

#Retest reliability
IE4_D$ILOC <- IE4_D$ILOC1+IE4_D$ILOC2
IE4_D$ELOC <- IE4_D$ELOC1+IE4_D$ELOC2
IE4_D$ILOCrt <- IE4_D$ILOC1rt+IE4_D$ILOC2rt
IE4_D$ELOCrt <- IE4_D$ELOC1rt+IE4_D$ELOC2rt
cor.test(IE4_D$ILOC, IE4_D$ILOCrt, use = "pairwise.complete.obs")
cor.test(IE4_D$ELOC, IE4_D$ELOCrt, use = "pairwise.complete.obs")

#Cronbach's alpha (BFI-2-XS and RSES)
alpha(subset(IE4_D, select = c(EXTR1R, EXTR2, EXTR3)))
alpha(subset(IE4_D, select = c(AGRE1, AGRE2R, AGRE3)))
alpha(subset(IE4_D, select = c(CONS1R, CONS2R, CONS3)))
alpha(subset(IE4_D, select = c(NEGA1, NEGA2, NEGA3R)))
alpha(subset(IE4_D, select = c(OPEN1, OPEN2R, OPEN3)))

alpha(subset(IE4_D,
```

```
        select = c(RSES1, RSES2R, RSES3, RSES4, RSES5R, RSES6R, RSES7, RSES8R, RSES9R, RSES10)))

#################################################################################

############################################
#Step 4: Construct and criterion validity
############################################

##UK

#Empty vectors to save correlations
UK_ILOC_cor <- c()
UK_ELOC_cor <- c()

#Name correlations
dimension.matrix <- list(c("EXTR", "AGRE", "CONS", "NEGA", "OPEN", "RSES",
                           "ASKU", "URGE", "PREM", "PERS", "SENS", "SOP",
                           "KUSIV3", "LISA1", "RISK1", "IPEF", "EPEF", "VICT",
                           "BENE", "OBSE", "OFFE", "SDPQ", "SDNQ", "EMPL.unempl",
                           "EMPL.retired", "INCO", "SCHO", "AGE", "SEX"),
                         c("UK", "DE"))

#BFI-2-XS
IE4_UK$EXTR <- IE4_UK$EXTR1R+IE4_UK$EXTR2+IE4_UK$EXTR3
IE4_UK$AGRE <- IE4_UK$AGRE1+IE4_UK$AGRE2R+IE4_UK$AGRE3
IE4_UK$CONS <- IE4_UK$CONS1R+IE4_UK$CONS2R+IE4_UK$CONS3
IE4_UK$NEGA <- IE4_UK$NEGA1+IE4_UK$NEGA2+IE4_UK$NEGA3R
IE4_UK$OPEN <- IE4_UK$OPEN1+IE4_UK$OPEN2R+IE4_UK$OPEN3

cor.test(IE4_UK$ILOC, IE4_UK$EXTR, use = "pairwise.complete.obs")
UK_ILOC_cor <- c(UK_ILOC_cor, cor.test(IE4_UK$ILOC, IE4_UK$EXTR, use = "pairwise.complete.obs")[["estimate"]][["cor"]])
cor.test(IE4_UK$ELOC, IE4_UK$EXTR, use = "pairwise.complete.obs")
UK_ELOC_cor <- c(UK_ELOC_cor, cor.test(IE4_UK$ELOC, IE4_UK$EXTR, use = "pairwise.complete.obs")[["estimate"]][["cor"]])

cor.test(IE4_UK$ILOC, IE4_UK$AGRE, use = "pairwise.complete.obs")
UK_ILOC_cor <- c(UK_ILOC_cor, cor.test(IE4_UK$ILOC, IE4_UK$AGRE, use = "pairwise.complete.obs")[["estimate"]][["cor"]])
cor.test(IE4_UK$ELOC, IE4_UK$AGRE, use = "pairwise.complete.obs")
UK_ELOC_cor <- c(UK_ELOC_cor, cor.test(IE4_UK$ELOC, IE4_UK$AGRE, use = "pairwise.complete.obs")[["estimate"]][["cor"]])
```

```
cor.test(IE4_UK$ILOC, IE4_UK$CONS, use = "pairwise.complete.obs")
UK_ILOC_cor <- c(UK_ILOC_cor, cor.test(IE4_UK$ILOC, IE4_UK$CONS, use =
"pairwise.complete.obs")[["estimate"]][["cor"]])
cor.test(IE4_UK$ELOC, IE4_UK$CONS, use = "pairwise.complete.obs")
UK_ELOC_cor <- c(UK_ELOC_cor, cor.test(IE4_UK$ELOC, IE4_UK$CONS, use =
"pairwise.complete.obs")[["estimate"]][["cor"]])

cor.test(IE4_UK$ILOC, IE4_UK$NEGA, use = "pairwise.complete.obs")
UK_ILOC_cor <- c(UK_ILOC_cor, cor.test(IE4_UK$ILOC, IE4_UK$NEGA, use =
"pairwise.complete.obs")[["estimate"]][["cor"]])
cor.test(IE4_UK$ELOC, IE4_UK$NEGA, use = "pairwise.complete.obs")
UK_ELOC_cor <- c(UK_ELOC_cor, cor.test(IE4_UK$ELOC, IE4_UK$NEGA, use =
"pairwise.complete.obs")[["estimate"]][["cor"]])

cor.test(IE4_UK$ILOC, IE4_UK$OPEN, use = "pairwise.complete.obs")
UK_ILOC_cor <- c(UK_ILOC_cor, cor.test(IE4_UK$ILOC, IE4_UK$OPEN, use =
"pairwise.complete.obs")[["estimate"]][["cor"]])
cor.test(IE4_UK$ELOC, IE4_UK$OPEN, use = "pairwise.complete.obs")
UK_ELOC_cor <- c(UK_ELOC_cor, cor.test(IE4_UK$ELOC, IE4_UK$OPEN, use =
"pairwise.complete.obs")[["estimate"]][["cor"]])

#RSES
IE4_UK$RSES <-
IE4_UK$RSES1+IE4_UK$RSES2R+IE4_UK$RSES3+IE4_UK$RSES4+IE4_UK$RSES5R
+IE4_UK$RSES6R+IE4_UK$RSES7+IE4_UK$RSES8R+IE4_UK$RSES9R+IE4_UK$RSE
S10

cor.test(IE4_UK$ILOC, IE4_UK$RSES, use = "pairwise.complete.obs")
UK_ILOC_cor <- c(UK_ILOC_cor, cor.test(IE4_UK$ILOC, IE4_UK$RSES, use =
"pairwise.complete.obs")[["estimate"]][["cor"]])
cor.test(IE4_UK$ELOC, IE4_UK$RSES, use = "pairwise.complete.obs")
UK_ELOC_cor <- c(UK_ELOC_cor, cor.test(IE4_UK$ELOC, IE4_UK$RSES, use =
"pairwise.complete.obs")[["estimate"]][["cor"]])

#GSE-3
IE4_UK$ASKU <- IE4_UK$ASKU1+IE4_UK$ASKU2+IE4_UK$ASKU3

cor.test(IE4_UK$ILOC, IE4_UK$ASKU, use = "pairwise.complete.obs")
UK_ILOC_cor <- c(UK_ILOC_cor, cor.test(IE4_UK$ILOC, IE4_UK$ASKU, use =
"pairwise.complete.obs")[["estimate"]][["cor"]])
cor.test(IE4_UK$ELOC, IE4_UK$ASKU, use = "pairwise.complete.obs")
```

```
UK_ELOC_cor <- c(UK_ELOC_cor, cor.test(IE4_UK$ELOC, IE4_UK$ASKU, use = "pairwise.complete.obs")[["estimate"]][["cor"]])

#I-8
IE4_UK$URGE <-IE4_UK$URGE1+IE4_UK$URGE2
IE4_UK$PREM <-IE4_UK$PREM1+IE4_UK$PREM2
IE4_UK$PERS <-IE4_UK$PERS1+IE4_UK$PERS2
IE4_UK$SENS <-IE4_UK$SENS1+IE4_UK$SENS2

cor.test(IE4_UK$ILOC, IE4_UK$URGE, use = "pairwise.complete.obs")
UK_ILOC_cor <- c(UK_ILOC_cor, cor.test(IE4_UK$ILOC, IE4_UK$URGE, use = "pairwise.complete.obs")[["estimate"]][["cor"]])
cor.test(IE4_UK$ELOC, IE4_UK$URGE, use = "pairwise.complete.obs")
UK_ELOC_cor <- c(UK_ELOC_cor, cor.test(IE4_UK$ELOC, IE4_UK$URGE, use = "pairwise.complete.obs")[["estimate"]][["cor"]])

cor.test(IE4_UK$ILOC, IE4_UK$PREM, use = "pairwise.complete.obs")
UK_ILOC_cor <- c(UK_ILOC_cor, cor.test(IE4_UK$ILOC, IE4_UK$PREM, use = "pairwise.complete.obs")[["estimate"]][["cor"]])
cor.test(IE4_UK$ELOC, IE4_UK$PREM, use = "pairwise.complete.obs")
UK_ELOC_cor <- c(UK_ELOC_cor, cor.test(IE4_UK$ELOC, IE4_UK$PREM, use = "pairwise.complete.obs")[["estimate"]][["cor"]])

cor.test(IE4_UK$ILOC, IE4_UK$PERS, use = "pairwise.complete.obs")
UK_ILOC_cor <- c(UK_ILOC_cor, cor.test(IE4_UK$ILOC, IE4_UK$PERS, use = "pairwise.complete.obs")[["estimate"]][["cor"]])
cor.test(IE4_UK$ELOC, IE4_UK$PERS, use = "pairwise.complete.obs")
UK_ELOC_cor <- c(UK_ELOC_cor, cor.test(IE4_UK$ELOC, IE4_UK$PERS, use = "pairwise.complete.obs")[["estimate"]][["cor"]])

cor.test(IE4_UK$ILOC, IE4_UK$SENS, use = "pairwise.complete.obs")
UK_ILOC_cor <- c(UK_ILOC_cor, cor.test(IE4_UK$ILOC, IE4_UK$SENS, use = "pairwise.complete.obs")[["estimate"]][["cor"]])
cor.test(IE4_UK$ELOC, IE4_UK$SENS, use = "pairwise.complete.obs")
UK_ELOC_cor <- c(UK_ELOC_cor, cor.test(IE4_UK$ELOC, IE4_UK$SENS, use = "pairwise.complete.obs")[["estimate"]][["cor"]])

#SOP2
IE4_UK$SOP <- IE4_UK$PESS1R + IE4_UK$OPTI1

cor.test(IE4_UK$ILOC, IE4_UK$SOP, use = "pairwise.complete.obs")
UK_ILOC_cor <- c(UK_ILOC_cor, cor.test(IE4_UK$ILOC, IE4_UK$SOP, use = "pairwise.complete.obs")[["estimate"]][["cor"]])
```

```
cor.test(IE4_UK$ELOC, IE4_UK$SOP, use = "pairwise.complete.obs")
UK_ELOC_cor <- c(UK_ELOC_cor, cor.test(IE4_UK$ELOC, IE4_UK$SOP, use =
"pairwise.complete.obs")[["estimate"]][["cor"]])

#KUSIV3
IE4_UK$KUSIV3 <-IE4_UK$KUSI1+IE4_UK$KUSI2R+IE4_UK$KUSI3

cor.test(IE4_UK$ILOC, IE4_UK$KUSIV3, use = "pairwise.complete.obs")
UK_ILOC_cor <- c(UK_ILOC_cor, cor.test(IE4_UK$ILOC, IE4_UK$KUSIV3, use =
"pairwise.complete.obs")[["estimate"]][["cor"]])
cor.test(IE4_UK$ELOC, IE4_UK$KUSIV3, use = "pairwise.complete.obs")
UK_ELOC_cor <- c(UK_ELOC_cor, cor.test(IE4_UK$ELOC, IE4_UK$KUSIV3, use =
"pairwise.complete.obs")[["estimate"]][["cor"]])

#L-1
cor.test(IE4_UK$ILOC, IE4_UK$LISA1, use = "pairwise.complete.obs")
UK_ILOC_cor <- c(UK_ILOC_cor, cor.test(IE4_UK$ILOC, IE4_UK$LISA1, use =
"pairwise.complete.obs")[["estimate"]][["cor"]])
cor.test(IE4_UK$ELOC, IE4_UK$LISA1, use = "pairwise.complete.obs")
UK_ELOC_cor <- c(UK_ELOC_cor, cor.test(IE4_UK$ELOC, IE4_UK$LISA1, use =
"pairwise.complete.obs")[["estimate"]][["cor"]])

#R-1
cor.test(IE4_UK$ILOC, IE4_UK$RISK1, use = "pairwise.complete.obs")
UK_ILOC_cor <- c(UK_ILOC_cor, cor.test(IE4_UK$ILOC, IE4_UK$RISK1, use =
"pairwise.complete.obs")[["estimate"]][["cor"]])
cor.test(IE4_UK$ELOC, IE4_UK$RISK1, use = "pairwise.complete.obs")
UK_ELOC_cor <- c(UK_ELOC_cor, cor.test(IE4_UK$ELOC, IE4_UK$RISK1, use =
"pairwise.complete.obs")[["estimate"]][["cor"]])

#PESS
IE4_UK$IPEF <- (IE4_UK$IPEF1+IE4_UK$IPEF2)/2
IE4_UK$EPEF <- (IE4_UK$EPEF1+IE4_UK$EPEF2)/2

cor.test(IE4_UK$ILOC, IE4_UK$IPEF, use = "pairwise.complete.obs")
UK_ILOC_cor <- c(UK_ILOC_cor, cor.test(IE4_UK$ILOC, IE4_UK$IPEF, use =
"pairwise.complete.obs")[["estimate"]][["cor"]])
cor.test(IE4_UK$ELOC, IE4_UK$IPEF, use = "pairwise.complete.obs")
UK_ELOC_cor <- c(UK_ELOC_cor, cor.test(IE4_UK$ELOC, IE4_UK$IPEF, use =
"pairwise.complete.obs")[["estimate"]][["cor"]])

cor.test(IE4_UK$ILOC, IE4_UK$EPEF, use = "pairwise.complete.obs")
```

```
UK_ILOC_cor <- c(UK_ILOC_cor, cor.test(IE4_UK$ILOC, IE4_UK$EPEF, use =
"pairwise.complete.obs")[["estimate"]][["cor"]])
cor.test(IE4_UK$ELOC, IE4_UK$EPEF, use = "pairwise.complete.obs")
UK_ELOC_cor <- c(UK_ELOC_cor, cor.test(IE4_UK$ELOC, IE4_UK$EPEF, use =
"pairwise.complete.obs")[["estimate"]][["cor"]])

#JSS-8
IE4_UK$VICT <- IE4_UK$VICT1+IE4_UK$VICT2
IE4_UK$OBSE <- IE4_UK$OBSE1+IE4_UK$OBSE2
IE4_UK$BENE <- IE4_UK$BENE1+IE4_UK$BENE2
IE4_UK$OFFE <- IE4_UK$OFFE1+IE4_UK$OFFE2

cor.test(IE4_UK$ILOC, IE4_UK$VICT, use = "pairwise.complete.obs")
UK_ILOC_cor <- c(UK_ILOC_cor, cor.test(IE4_UK$ILOC, IE4_UK$VICT, use =
"pairwise.complete.obs")[["estimate"]][["cor"]])
cor.test(IE4_UK$ELOC, IE4_UK$VICT, use = "pairwise.complete.obs")
UK_ELOC_cor <- c(UK_ELOC_cor, cor.test(IE4_UK$ELOC, IE4_UK$VICT, use =
"pairwise.complete.obs")[["estimate"]][["cor"]])

cor.test(IE4_UK$ILOC, IE4_UK$OBSE, use = "pairwise.complete.obs")
UK_ILOC_cor <- c(UK_ILOC_cor, cor.test(IE4_UK$ILOC, IE4_UK$OBSE, use =
"pairwise.complete.obs")[["estimate"]][["cor"]])
cor.test(IE4_UK$ELOC, IE4_UK$OBSE, use = "pairwise.complete.obs")
UK_ELOC_cor <- c(UK_ELOC_cor, cor.test(IE4_UK$ELOC, IE4_UK$OBSE, use =
"pairwise.complete.obs")[["estimate"]][["cor"]])

cor.test(IE4_UK$ILOC, IE4_UK$BENE, use = "pairwise.complete.obs")
UK_ILOC_cor <- c(UK_ILOC_cor, cor.test(IE4_UK$ILOC, IE4_UK$BENE, use =
"pairwise.complete.obs")[["estimate"]][["cor"]])
cor.test(IE4_UK$ELOC, IE4_UK$BENE, use = "pairwise.complete.obs")
UK_ELOC_cor <- c(UK_ELOC_cor, cor.test(IE4_UK$ELOC, IE4_UK$BENE, use =
"pairwise.complete.obs")[["estimate"]][["cor"]])

cor.test(IE4_UK$ILOC, IE4_UK$OFFE, use = "pairwise.complete.obs")
UK_ILOC_cor <- c(UK_ILOC_cor, cor.test(IE4_UK$ILOC, IE4_UK$OFFE, use =
"pairwise.complete.obs")[["estimate"]][["cor"]])
cor.test(IE4_UK$ELOC, IE4_UK$OFFE, use = "pairwise.complete.obs")
UK_ELOC_cor <- c(UK_ELOC_cor, cor.test(IE4_UK$ELOC, IE4_UK$OFFE, use =
"pairwise.complete.obs")[["estimate"]][["cor"]])

#KSE-G
IE4_UK$SDPQ <- IE4_UK$SDPQ1+IE4_UK$SDPQ2+IE4_UK$SDPQ3
IE4_UK$SDNQ <- IE4_UK$SDNQ1+IE4_UK$SDNQ2+IE4_UK$SDNQ3
```

```
cor.test(IE4_UK$ILOC, IE4_UK$SDPQ, use = "pairwise.complete.obs")
UK_ILOC_cor <- c(UK_ILOC_cor, cor.test(IE4_UK$ILOC, IE4_UK$SDPQ, use =
"pairwise.complete.obs")[["estimate"]][["cor"]])
cor.test(IE4_UK$ELOC, IE4_UK$SDPQ, use = "pairwise.complete.obs")
UK_ELOC_cor <- c(UK_ELOC_cor, cor.test(IE4_UK$ELOC, IE4_UK$SDPQ, use =
"pairwise.complete.obs")[["estimate"]][["cor"]])

cor.test(IE4_UK$ILOC, IE4_UK$SDNQ, use = "pairwise.complete.obs")
UK_ILOC_cor <- c(UK_ILOC_cor, cor.test(IE4_UK$ILOC, IE4_UK$SDNQ, use =
"pairwise.complete.obs")[["estimate"]][["cor"]])
cor.test(IE4_UK$ELOC, IE4_UK$SDNQ, use = "pairwise.complete.obs")
UK_ELOC_cor <- c(UK_ELOC_cor, cor.test(IE4_UK$ELOC, IE4_UK$SDNQ, use =
"pairwise.complete.obs")[["estimate"]][["cor"]])

#Employment status
#1) employed
#2) self-employed
#3) out of work and looking for work
#4) out of work but not currently looking for work
#5) doing housework
#6) pupil/student
#7) apprentice/internship
#8) retired
#[9) none of what is mentioned above]
describe(IE4_UK$EMPL)
#unemployed vs. (self-)employed
IE4_UK$EMPL.unempl <- recode(IE4_UK$EMPL, "3:4 = 2; 1:2 = 1; else = NA")
describe(IE4_UK$EMPL.unempl)
#retired/doing housework vs. (self-)employed
IE4_UK$EMPL.retired <- recode(IE4_UK$EMPL, "5 = 2; 8 = 2; 1:2 = 1; else = NA")
describe(IE4_UK$EMPL.retired)

cor.test(IE4_UK$ILOC, IE4_UK$EMPL.unempl, use = "pairwise.complete.obs")
UK_ILOC_cor <- c(UK_ILOC_cor, cor.test(IE4_UK$ILOC, IE4_UK$EMPL.unempl, use =
"pairwise.complete.obs")[["estimate"]][["cor"]])
cor.test(IE4_UK$ILOC, IE4_UK$EMPL.retired, use = "pairwise.complete.obs")
UK_ILOC_cor <- c(UK_ILOC_cor, cor.test(IE4_UK$ILOC, IE4_UK$EMPL.retired, use =
"pairwise.complete.obs")[["estimate"]][["cor"]])

cor.test(IE4_UK$ELOC, IE4_UK$EMPL.unempl, use = "pairwise.complete.obs")
UK_ELOC_cor <- c(UK_ELOC_cor, cor.test(IE4_UK$ELOC, IE4_UK$EMPL.unempl, use
= "pairwise.complete.obs")[["estimate"]][["cor"]])
```

```
cor.test(IE4_UK$ELOC, IE4_UK$EMPL.retired, use = "pairwise.complete.obs")
UK_ELOC_cor <- c(UK_ELOC_cor, cor.test(IE4_UK$ELOC, IE4_UK$EMPL.retired, use =
"pairwise.complete.obs")[["estimate"]][["cor"]])

#Income
cor.test(IE4_UK$ILOC, IE4_UK$INCO, use = "pairwise.complete.obs")
UK_ILOC_cor <- c(UK_ILOC_cor, cor.test(IE4_UK$ILOC, IE4_UK$INCO, use =
"pairwise.complete.obs")[["estimate"]][["cor"]])
cor.test(IE4_UK$ELOC, IE4_UK$INCO, use = "pairwise.complete.obs")
UK_ELOC_cor <- c(UK_ELOC_cor, cor.test(IE4_UK$ELOC, IE4_UK$INCO, use =
"pairwise.complete.obs")[["estimate"]][["cor"]])

#Education
cor.test(IE4_UK$ILOC, IE4_UK$SCHO, use = "pairwise.complete.obs")
UK_ILOC_cor <- c(UK_ILOC_cor, cor.test(IE4_UK$ILOC, IE4_UK$SCHO, use =
"pairwise.complete.obs")[["estimate"]][["cor"]])
cor.test(IE4_UK$ELOC, IE4_UK$SCHO, use = "pairwise.complete.obs")
UK_ELOC_cor <- c(UK_ELOC_cor, cor.test(IE4_UK$ELOC, IE4_UK$SCHO, use =
"pairwise.complete.obs")[["estimate"]][["cor"]])

#Age
cor.test(IE4_UK$ILOC, IE4_UK$AGE, use = "pairwise.complete.obs")
UK_ILOC_cor <- c(UK_ILOC_cor, cor.test(IE4_UK$ILOC, IE4_UK$AGE, use =
"pairwise.complete.obs")[["estimate"]][["cor"]])
cor.test(IE4_UK$ELOC, IE4_UK$AGE, use = "pairwise.complete.obs")
UK_ELOC_cor <- c(UK_ELOC_cor, cor.test(IE4_UK$ELOC, IE4_UK$AGE, use =
"pairwise.complete.obs")[["estimate"]][["cor"]])

#Gender
cor.test(IE4_UK$ILOC, IE4_UK$SEX, use = "pairwise.complete.obs")
UK_ILOC_cor <- c(UK_ILOC_cor, cor.test(IE4_UK$ILOC, IE4_UK$SEX, use =
"pairwise.complete.obs")[["estimate"]][["cor"]])
cor.test(IE4_UK$ELOC, IE4_UK$SEX, use = "pairwise.complete.obs")
UK_ELOC_cor <- c(UK_ELOC_cor, cor.test(IE4_UK$ELOC, IE4_UK$SEX, use =
"pairwise.complete.obs")[["estimate"]][["cor"]])

################################################################################

##Germany

#Empty vectors to save correlations
D_ILOC_cor <- c()
D_ELOC_cor <- c()
```

```
#Name correlations
dimension.matrix <- list(c("EXTR", "AGRE", "CONS", "NEGA", "OPEN", "RSES",
                           "ASKU", "URGE", "PREM", "PERS", "SENS", "SOP",
                           "KUSIV3", "LISA1", "RISK1", "IPEF", "EPEF", "VICT",
                           "BENE", "OBSE", "OFFE", "SDPQ", "SDNQ", "EMPL.unempl",
                           "EMPL.retired", "INCO", "SCHO", "AGE", "SEX"),
                         c("UK", "DE"))

#BFI-2-XS
IE4_D$EXTR <- IE4_D$EXTR1R+IE4_D$EXTR2+IE4_D$EXTR3
IE4_D$AGRE <- IE4_D$AGRE1+IE4_D$AGRE2R+IE4_D$AGRE3
IE4_D$CONS <- IE4_D$CONS1R+IE4_D$CONS2R+IE4_D$CONS3
IE4_D$NEGA <- IE4_D$NEGA1+IE4_D$NEGA2+IE4_D$NEGA3R
IE4_D$OPEN <- IE4_D$OPEN1+IE4_D$OPEN2R+IE4_D$OPEN3

cor.test(IE4_D$ILOC, IE4_D$EXTR, use = "pairwise.complete.obs")
D_ILOC_cor <- c(D_ILOC_cor, cor.test(IE4_D$ILOC, IE4_D$EXTR, use =
"pairwise.complete.obs")[["estimate"]][["cor"]])
cor.test(IE4_D$ELOC, IE4_D$EXTR, use = "pairwise.complete.obs")
D_ELOC_cor <- c(D_ELOC_cor, cor.test(IE4_D$ELOC, IE4_D$EXTR, use =
"pairwise.complete.obs")[["estimate"]][["cor"]])

cor.test(IE4_D$ILOC, IE4_D$AGRE, use = "pairwise.complete.obs")
D_ILOC_cor <- c(D_ILOC_cor, cor.test(IE4_D$ILOC, IE4_D$AGRE, use =
"pairwise.complete.obs")[["estimate"]][["cor"]])
cor.test(IE4_D$ELOC, IE4_D$AGRE, use = "pairwise.complete.obs")
D_ELOC_cor <- c(D_ELOC_cor, cor.test(IE4_D$ELOC, IE4_D$AGRE, use =
"pairwise.complete.obs")[["estimate"]][["cor"]])

cor.test(IE4_D$ILOC, IE4_D$CONS, use = "pairwise.complete.obs")
D_ILOC_cor <- c(D_ILOC_cor, cor.test(IE4_D$ILOC, IE4_D$CONS, use =
"pairwise.complete.obs")[["estimate"]][["cor"]])
cor.test(IE4_D$ELOC, IE4_D$CONS, use = "pairwise.complete.obs")
D_ELOC_cor <- c(D_ELOC_cor, cor.test(IE4_D$ELOC, IE4_D$AGRE, use =
"pairwise.complete.obs")[["estimate"]][["cor"]])

cor.test(IE4_D$ILOC, IE4_D$NEGA, use = "pairwise.complete.obs")
D_ILOC_cor <- c(D_ILOC_cor, cor.test(IE4_D$ILOC, IE4_D$NEGA, use =
"pairwise.complete.obs")[["estimate"]][["cor"]])
cor.test(IE4_D$ELOC, IE4_D$NEGA, use = "pairwise.complete.obs")
D_ELOC_cor <- c(D_ELOC_cor, cor.test(IE4_D$ELOC, IE4_D$NEGA, use =
"pairwise.complete.obs")[["estimate"]][["cor"]])
```

```
cor.test(IE4_D$ILOC, IE4_D$OPEN, use = "pairwise.complete.obs")
D_ILOC_cor <- c(D_ILOC_cor, cor.test(IE4_D$ILOC, IE4_D$OPEN, use =
"pairwise.complete.obs")[["estimate"]][["cor"]])
cor.test(IE4_D$ELOC, IE4_D$OPEN, use = "pairwise.complete.obs")
D_ELOC_cor <- c(D_ELOC_cor, cor.test(IE4_D$ELOC, IE4_D$OPEN, use =
"pairwise.complete.obs")[["estimate"]][["cor"]])

#RSES
IE4_D$RSES <-
IE4_D$RSES1+IE4_D$RSES2R+IE4_D$RSES3+IE4_D$RSES4+IE4_D$RSES5R+IE4_D$
RSES6R+IE4_D$RSES7+IE4_D$RSES8R+IE4_D$RSES9R+IE4_D$RSES10

cor.test(IE4_D$ILOC, IE4_D$RSES, use = "pairwise.complete.obs")
D_ILOC_cor <- c(D_ILOC_cor, cor.test(IE4_D$ILOC, IE4_D$RSES, use =
"pairwise.complete.obs")[["estimate"]][["cor"]])
cor.test(IE4_D$ELOC, IE4_D$RSES, use = "pairwise.complete.obs")
D_ELOC_cor <- c(D_ELOC_cor, cor.test(IE4_D$ELOC, IE4_D$RSES, use =
"pairwise.complete.obs")[["estimate"]][["cor"]])

#ASKU
IE4_D$ASKU <- IE4_D$ASKU1+IE4_D$ASKU2+IE4_D$ASKU3

cor.test(IE4_D$ILOC, IE4_D$ASKU, use = "pairwise.complete.obs")
D_ILOC_cor <- c(D_ILOC_cor, cor.test(IE4_D$ILOC, IE4_D$ASKU, use =
"pairwise.complete.obs")[["estimate"]][["cor"]])
cor.test(IE4_D$ELOC, IE4_D$ASKU, use = "pairwise.complete.obs")
D_ELOC_cor <- c(D_ELOC_cor, cor.test(IE4_D$ELOC, IE4_D$ASKU, use =
"pairwise.complete.obs")[["estimate"]][["cor"]])

#I-8
IE4_D$URGE <-IE4_D$URGE1+IE4_D$URGE2
IE4_D$PREM <-IE4_D$PREM1+IE4_D$PREM2
IE4_D$PERS <-IE4_D$PERS1+IE4_D$PERS2
IE4_D$SENS <-IE4_D$SENS1+IE4_D$SENS2

cor.test(IE4_D$ILOC, IE4_D$URGE, use = "pairwise.complete.obs")
D_ILOC_cor <- c(D_ILOC_cor, cor.test(IE4_D$ILOC, IE4_D$URGE, use =
"pairwise.complete.obs")[["estimate"]][["cor"]])
cor.test(IE4_D$ELOC, IE4_D$URGE, use = "pairwise.complete.obs")
D_ELOC_cor <- c(D_ELOC_cor, cor.test(IE4_D$ELOC, IE4_D$URGE, use =
"pairwise.complete.obs")[["estimate"]][["cor"]])
```

```
cor.test(IE4_D$ILOC, IE4_D$PREM, use = "pairwise.complete.obs")
D_ILOC_cor <- c(D_ILOC_cor, cor.test(IE4_D$ILOC, IE4_D$PREM, use =
"pairwise.complete.obs")[["estimate"]][["cor"]])
cor.test(IE4_D$ELOC, IE4_D$PREM, use = "pairwise.complete.obs")
D_ELOC_cor <- c(D_ELOC_cor, cor.test(IE4_D$ELOC, IE4_D$PREM, use =
"pairwise.complete.obs")[["estimate"]][["cor"]])

cor.test(IE4_D$ILOC, IE4_D$PERS, use = "pairwise.complete.obs")
D_ILOC_cor <- c(D_ILOC_cor, cor.test(IE4_D$ILOC, IE4_D$PERS, use =
"pairwise.complete.obs")[["estimate"]][["cor"]])
cor.test(IE4_D$ELOC, IE4_D$PERS, use = "pairwise.complete.obs")
D_ELOC_cor <- c(D_ELOC_cor, cor.test(IE4_D$ELOC, IE4_D$PERS, use =
"pairwise.complete.obs")[["estimate"]][["cor"]])

cor.test(IE4_D$ILOC, IE4_D$SENS, use = "pairwise.complete.obs")
D_ILOC_cor <- c(D_ILOC_cor, cor.test(IE4_D$ILOC, IE4_D$SENS, use =
"pairwise.complete.obs")[["estimate"]][["cor"]])
cor.test(IE4_D$ELOC, IE4_D$SENS, use = "pairwise.complete.obs")
D_ELOC_cor <- c(D_ELOC_cor, cor.test(IE4_D$ELOC, IE4_D$SENS, use =
"pairwise.complete.obs")[["estimate"]][["cor"]])

#SOP2
IE4_D$SOP <- IE4_D$PESS1R + IE4_D$OPTI1

cor.test(IE4_D$ILOC, IE4_D$SOP, use = "pairwise.complete.obs")
D_ILOC_cor <- c(D_ILOC_cor, cor.test(IE4_D$ILOC, IE4_D$SOP, use =
"pairwise.complete.obs")[["estimate"]][["cor"]])
cor.test(IE4_D$ELOC, IE4_D$SOP, use = "pairwise.complete.obs")
D_ELOC_cor <- c(D_ELOC_cor, cor.test(IE4_D$ELOC, IE4_D$SOP, use =
"pairwise.complete.obs")[["estimate"]][["cor"]])

#KUSIV3
IE4_D$KUSIV3 <-IE4_D$KUSI1+IE4_D$KUSI2R+IE4_D$KUSI3

cor.test(IE4_D$ILOC, IE4_D$KUSIV3, use = "pairwise.complete.obs")
D_ILOC_cor <- c(D_ILOC_cor, cor.test(IE4_D$ILOC, IE4_D$KUSIV3, use =
"pairwise.complete.obs")[["estimate"]][["cor"]])
cor.test(IE4_D$ELOC, IE4_D$KUSIV3, use = "pairwise.complete.obs")
D_ELOC_cor <- c(D_ELOC_cor, cor.test(IE4_D$ELOC, IE4_D$KUSIV3, use =
"pairwise.complete.obs")[["estimate"]][["cor"]])

#L-1
cor.test(IE4_D$ILOC, IE4_D$LISA1, use = "pairwise.complete.obs")
```

```
D_ILOC_cor <- c(D_ILOC_cor, cor.test(IE4_D$ILOC, IE4_D$LISA1, use =
"pairwise.complete.obs")[["estimate"]][["cor"]])
cor.test(IE4_D$ELOC, IE4_D$LISA1, use = "pairwise.complete.obs")
D_ELOC_cor <- c(D_ELOC_cor, cor.test(IE4_D$ELOC, IE4_D$LISA1, use =
"pairwise.complete.obs")[["estimate"]][["cor"]])

#R-1
cor.test(IE4_D$ILOC, IE4_D$RISK1, use = "pairwise.complete.obs")
D_ILOC_cor <- c(D_ILOC_cor, cor.test(IE4_D$ILOC, IE4_D$RISK1, use =
"pairwise.complete.obs")[["estimate"]][["cor"]])
cor.test(IE4_D$ELOC, IE4_D$RISK1, use = "pairwise.complete.obs")
D_ELOC_cor <- c(D_ELOC_cor, cor.test(IE4_D$ELOC, IE4_D$RISK1, use =
"pairwise.complete.obs")[["estimate"]][["cor"]])

#PEKS
IE4_D$IPEF <- (IE4_D$IPEF1+IE4_D$IPEF2)/2
IE4_D$EPEF <- (IE4_D$EPEF1+IE4_D$EPEF2)/2

cor.test(IE4_D$ILOC, IE4_D$IPEF, use = "pairwise.complete.obs")
D_ILOC_cor <- c(D_ILOC_cor, cor.test(IE4_D$ILOC, IE4_D$IPEF, use =
"pairwise.complete.obs")[["estimate"]][["cor"]])
cor.test(IE4_D$ELOC, IE4_D$IPEF, use = "pairwise.complete.obs")
D_ELOC_cor <- c(D_ELOC_cor, cor.test(IE4_D$ELOC, IE4_D$IPEF, use =
"pairwise.complete.obs")[["estimate"]][["cor"]])

cor.test(IE4_D$ILOC, IE4_D$EPEF, use = "pairwise.complete.obs")
D_ILOC_cor <- c(D_ILOC_cor, cor.test(IE4_D$ILOC, IE4_D$EPEF, use =
"pairwise.complete.obs")[["estimate"]][["cor"]])
cor.test(IE4_D$ELOC, IE4_D$EPEF, use = "pairwise.complete.obs")
D_ELOC_cor <- c(D_ELOC_cor, cor.test(IE4_D$ELOC, IE4_D$EPEF, use =
"pairwise.complete.obs")[["estimate"]][["cor"]])

#USS-8
IE4_D$VICT <- IE4_D$VICT1+IE4_D$VICT2
IE4_D$OBSE <- IE4_D$OBSE1+IE4_D$OBSE2
IE4_D$BENE <- IE4_D$BENE1+IE4_D$BENE2
IE4_D$OFFE <- IE4_D$OFFE1+IE4_D$OFFE2

cor.test(IE4_D$ILOC, IE4_D$VICT, use = "pairwise.complete.obs")
D_ILOC_cor <- c(D_ILOC_cor, cor.test(IE4_D$ILOC, IE4_D$VICT, use =
"pairwise.complete.obs")[["estimate"]][["cor"]])
cor.test(IE4_D$ELOC, IE4_D$VICT, use = "pairwise.complete.obs")
```

```
D_ELOC_cor <- c(D_ELOC_cor, cor.test(IE4_D$ELOC, IE4_D$VICT, use =
"pairwise.complete.obs")[["estimate"]][["cor"]])

cor.test(IE4_D$ILOC, IE4_D$OBSE, use = "pairwise.complete.obs")
D_ILOC_cor <- c(D_ILOC_cor, cor.test(IE4_D$ILOC, IE4_D$OBSE, use =
"pairwise.complete.obs")[["estimate"]][["cor"]])
cor.test(IE4_D$ELOC, IE4_D$OBSE, use = "pairwise.complete.obs")
D_ELOC_cor <- c(D_ELOC_cor, cor.test(IE4_D$ELOC, IE4_D$OBSE, use =
"pairwise.complete.obs")[["estimate"]][["cor"]])

cor.test(IE4_D$ILOC, IE4_D$BENE, use = "pairwise.complete.obs")
D_ILOC_cor <- c(D_ILOC_cor, cor.test(IE4_D$ILOC, IE4_D$BENE, use =
"pairwise.complete.obs")[["estimate"]][["cor"]])
cor.test(IE4_D$ELOC, IE4_D$BENE, use = "pairwise.complete.obs")
D_ELOC_cor <- c(D_ELOC_cor, cor.test(IE4_D$ELOC, IE4_D$BENE, use =
"pairwise.complete.obs")[["estimate"]][["cor"]])

cor.test(IE4_D$ILOC, IE4_D$OFFE, use = "pairwise.complete.obs")
D_ILOC_cor <- c(D_ILOC_cor, cor.test(IE4_D$ILOC, IE4_D$OFFE, use =
"pairwise.complete.obs")[["estimate"]][["cor"]])
cor.test(IE4_D$ELOC, IE4_D$OFFE, use = "pairwise.complete.obs")
D_ELOC_cor <- c(D_ELOC_cor, cor.test(IE4_D$ELOC, IE4_D$OFFE, use =
"pairwise.complete.obs")[["estimate"]][["cor"]])

#KSE-G
IE4_D$SDPQ <- IE4_D$SDPQ1+IE4_D$SDPQ2+IE4_D$SDPQ3
IE4_D$SDNQ <- IE4_D$SDNQ1+IE4_D$SDNQ2+IE4_D$SDNQ3

cor.test(IE4_D$ILOC, IE4_D$SDPQ, use = "pairwise.complete.obs")
D_ILOC_cor <- c(D_ILOC_cor, cor.test(IE4_D$ILOC, IE4_D$SDPQ, use =
"pairwise.complete.obs")[["estimate"]][["cor"]])
cor.test(IE4_D$ELOC, IE4_D$SDPQ, use = "pairwise.complete.obs")
D_ELOC_cor <- c(D_ELOC_cor, cor.test(IE4_D$ELOC, IE4_D$SDPQ, use =
"pairwise.complete.obs")[["estimate"]][["cor"]])

cor.test(IE4_D$ILOC, IE4_D$SDNQ, use = "pairwise.complete.obs")
D_ILOC_cor <- c(D_ILOC_cor, cor.test(IE4_D$ILOC, IE4_D$SDNQ, use =
"pairwise.complete.obs")[["estimate"]][["cor"]])
cor.test(IE4_D$ELOC, IE4_D$SDNQ, use = "pairwise.complete.obs")
D_ELOC_cor <- c(D_ELOC_cor, cor.test(IE4_D$ELOC, IE4_D$SDNQ, use =
"pairwise.complete.obs")[["estimate"]][["cor"]])

#Employment status
```

```
#1) employed
#2) self-employed
#3) out of work and looking for work
#4) out of work but not currently looking for work
#5) doing housework
#6) pupil/student
#7) apprentice/internship
#8) retired
#[9) none of what is mentioned above]
describe(IE4_D$EMPL)
#unemployed vs. employed
IE4_D$EMPL.unempl <- recode(IE4_D$EMPL, "3:4 = 2; 1:2 = 1; else = NA")
describe(IE4_D$EMPL.unempl)
#retrired/doing housework vs. employed
IE4_D$EMPL.retired <- recode(IE4_D$EMPL, "5 = 2; 8 = 2; 1:2 = 1; else = NA")
describe(IE4_D$EMPL.retired)

cor.test(IE4_D$ILOC, IE4_D$EMPL.unempl, use = "pairwise.complete.obs")
D_ILOC_cor <- c(D_ILOC_cor, cor.test(IE4_D$ILOC, IE4_D$EMPL.unempl, use =
"pairwise.complete.obs")[["estimate"]][["cor"]])
cor.test(IE4_D$ILOC, IE4_D$EMPL.retired, use = "pairwise.complete.obs")
D_ILOC_cor <- c(D_ILOC_cor, cor.test(IE4_D$ILOC, IE4_D$EMPL.retired, use =
"pairwise.complete.obs")[["estimate"]][["cor"]])

cor.test(IE4_D$ELOC, IE4_D$EMPL.unempl, use = "pairwise.complete.obs")
D_ELOC_cor <- c(D_ELOC_cor, cor.test(IE4_D$ELOC, IE4_D$EMPL.unempl, use =
"pairwise.complete.obs")[["estimate"]][["cor"]])
cor.test(IE4_D$ELOC, IE4_D$EMPL.retired, use = "pairwise.complete.obs")
D_ELOC_cor <- c(D_ELOC_cor, cor.test(IE4_D$ELOC, IE4_D$EMPL.retired, use =
"pairwise.complete.obs")[["estimate"]][["cor"]])

#Income
cor.test(IE4_D$ILOC, IE4_D$INCO, use = "pairwise.complete.obs")
D_ILOC_cor <- c(D_ILOC_cor, cor.test(IE4_D$ILOC, IE4_D$INCO, use =
"pairwise.complete.obs")[["estimate"]][["cor"]])
cor.test(IE4_D$ELOC, IE4_D$INCO, use = "pairwise.complete.obs")
D_ELOC_cor <- c(D_ELOC_cor, cor.test(IE4_D$ELOC, IE4_D$INCO, use =
"pairwise.complete.obs")[["estimate"]][["cor"]])

#Education
cor.test(IE4_D$ILOC, IE4_D$SCHO, use = "pairwise.complete.obs")
D_ILOC_cor <- c(D_ILOC_cor, cor.test(IE4_D$ILOC, IE4_D$SCHO, use =
"pairwise.complete.obs")[["estimate"]][["cor"]])
```

```
cor.test(IE4_D$ELOC, IE4_D$SCHO, use = "pairwise.complete.obs")
D_ELOC_cor <- c(D_ELOC_cor, cor.test(IE4_D$ELOC, IE4_D$SCHO, use =
"pairwise.complete.obs")[["estimate"]][["cor"]])

#Age
cor.test(IE4_D$ILOC, IE4_D$AGE, use = "pairwise.complete.obs")
D_ILOC_cor <- c(D_ILOC_cor, cor.test(IE4_D$ILOC, IE4_D$AGE, use =
"pairwise.complete.obs")[["estimate"]][["cor"]])
cor.test(IE4_D$ELOC, IE4_D$AGE, use = "pairwise.complete.obs")
D_ELOC_cor <- c(D_ELOC_cor, cor.test(IE4_D$ELOC, IE4_D$AGE, use =
"pairwise.complete.obs")[["estimate"]][["cor"]])

#Gender
cor.test(IE4_D$ILOC, IE4_D$SEX, use = "pairwise.complete.obs")
D_ILOC_cor <- c(D_ILOC_cor, cor.test(IE4_D$ILOC, IE4_D$SEX, use =
"pairwise.complete.obs")[["estimate"]][["cor"]])
cor.test(IE4_D$ELOC, IE4_D$SEX, use = "pairwise.complete.obs")
D_ELOC_cor <- c(D_ELOC_cor, cor.test(IE4_D$ELOC, IE4_D$SEX, use =
"pairwise.complete.obs")[["estimate"]][["cor"]])

################################################################################

##Comparison of correlation patterns across countries
ILOC.cor <- data.frame(matrix(data = c(UK_ILOC_cor,D_ILOC_cor),
                         nrow = length(dimension.matrix[[1]]),
                         ncol = 2, dimnames = dimension.matrix))
cor.test(ILOC.cor$UK, ILOC.cor$D, use = "pairwise.complete.obs")

ELOC.cor <- data.frame(matrix(data = c(UK_ELOC_cor,D_ELOC_cor),
                         nrow = length(dimension.matrix[[1]]),
                         ncol = 2, dimnames = dimension.matrix))
cor.test(ELOC.cor$UK, ELOC.cor$D, use = "pairwise.complete.obs")

################################################################################

##################################
#Step 5: Measurement invariance
##################################

##Two-dimensional model

#Metric invariance
```

```
IE4.fit1 <- cfa(IE4_MM_tau, data = IE4, group = "COUN", estimator = "mlr", missing =
"fiml", group.equal = "loadings")
summary(IE4.fit1, standardized = T, fit.measures = T)

#Scalar invariance
IE4.fit2 <- cfa(IE4_MM_tau, data = IE4, group = "COUN", estimator = "mlr", missing =
"fiml", group.equal = c("loadings", "intercepts"))
summary(IE4.fit2, standardized = T, fit.measures = T)
anova(IE4.fit2, IE4.fit1)

##########################################################################

##MIMIC model

#Essentially tau-equivalent model (metric multi-group model)
IE4_MM <- 'LV_ILOC =~ c(a1, a1)*ILOC1 + c(a1, a1)*ILOC2
        LV_ELOC =~ c(a2, a2)*ELOC1 + c(a2, a2)*ELOC2
        ILOC1 + ELOC1 ~ c(0, 0)*1
        LV_ILOC + LV_ELOC ~ NA*1'

IE4_MM.fit <- cfa(IE4_MM, data = IE4, group = "COUN", estimator = "mlr", missing =
"fiml", std.lv = FALSE)
summary(IE4_MM.fit, standardized = TRUE, fit.measures = TRUE)
#--> invariant item: ILOC2 (negative intercept in Germany, positive intercept in the UK)

#MIMIC model (scalar single-group model)
#Regression of latent variables and non-invariant item on country
IE4_MIMIC <- 'LV_ILOC =~ c(a1)*ILOC1 + c(a1)*ILOC2
          LV_ELOC =~ c(a2)*ELOC1 + c(a2)*ELOC2
          ILOC1 + ELOC1 ~ 0*1
          ILOC2 ~ c1*1
          ELOC2 ~ c2*1
          LV_ILOC + LV_ELOC ~ NA*1
          LV_ILOC + LV_ELOC + ILOC2 ~ COUN'

IE4_MIMIC.fit <- cfa(IE4_MIMIC, data = IE4, estimator = "mlr", missing = "fiml", std.lv =
FALSE)
summary(IE4_MIMIC.fit, standardized = TRUE, fit.measures = TRUE)

##########################################################################

##########################
#Step 6: Reference values
```

```
#########################

#Quote 1: male, lower education, 18-29
#Quote 2: male, lower education, 30-49
#Quote 3: male, lower education, 50-69
#Quote 4: male, middle education, 18-29
#Quote 5: male, middle education, 30-49
#Quote 6: male, middle education, 50-69
#Quote 7: male, upper education, 18-29
#Quote 8: male, upper education, 30-49
#Quote 9: male, upper education, 50-69
#Quote 10: female, lower education, 18-29
#Quote 11: female, lower education, 30-49
#Quote 12: female, lower education, 50-69
#Quote 13: female, middle education, 18-29
#Quote 14: female, middle education, 30-49
#Quote 15: female, middle education, 50-69
#Quote 16: female, upper education, 18-29
#Quote 17: female, upper education, 30-49
#Quote 18: female, upper education, 50-69

##UK

IE4_UK$ILOC <- (IE4_UK$ILOC1+IE4_UK$ILOC2)/2
IE4_UK$ELOC <- (IE4_UK$ELOC1+IE4_UK$ELOC2)/2
describe(IE4_UK$ILOC)
describe(IE4_UK$ELOC)

#Internal locus of control
tapply(IE4_UK$ILOC, IE4_UK$SEX, describe)
AGE1_UK <- subset(IE4_UK, AGE == 18 | AGE == 19 | AGE == 20 | AGE == 21 | AGE == 22 | AGE == 23 | AGE == 24 | AGE == 25 | AGE == 26
                | AGE == 27 | AGE == 28 | AGE == 29)
AGE2_UK <- subset(IE4_UK, AGE == 30 | AGE == 31 | AGE == 32 | AGE == 33 | AGE == 34 | AGE == 35 | AGE == 36 | AGE == 37 | AGE == 38
                | AGE == 39 | AGE == 40 | AGE == 41 | AGE == 42 | AGE == 43 | AGE == 44 | AGE == 45 | AGE == 46 | AGE == 47
                | AGE == 48 | AGE == 49)
AGE3_UK <- subset(IE4_UK, AGE == 50 | AGE == 51 | AGE == 52 | AGE == 53 | AGE == 54 | AGE == 55 | AGE == 56 | AGE == 57 | AGE == 58
                | AGE == 59 | AGE == 60 | AGE == 61 | AGE == 62 | AGE == 63 | AGE == 64 | AGE == 65 | AGE == 66 | AGE == 67
                | AGE == 68 | AGE == 69)
```

```
AGE1_UK$ILOC <- (AGE1_UK$ILOC1+AGE1_UK$ILOC2)/2
describe(AGE1_UK$ILOC)

AGE2_UK$ILOC <- (AGE2_UK$ILOC1+AGE2_UK$ILOC2)/2
describe(AGE2_UK$ILOC)

AGE3_UK$ILOC <- (AGE3_UK$ILOC1+AGE3_UK$ILOC2)/2
describe(AGE3_UK$ILOC)

#External locus of control
tapply(IE4_UK$ELOC, IE4_UK$SEX, describe)

AGE1_UK$ELOC <- (AGE1_UK$ELOC1+AGE1_UK$ELOC2)/2
describe(AGE1_UK$ELOC)

AGE2_UK$ELOC <- (AGE2_UK$ELOC1+AGE2_UK$ELOC2)/2
describe(AGE2_UK$ELOC)

AGE3_UK$ELOC <- (AGE3_UK$ELOC1+AGE3_UK$ELOC2)/2
describe(AGE3_UK$ELOC)

################################################################################

##Germany

IE4_D$ILOC <- (IE4_D$ILOC1+IE4_D$ILOC2)/2
IE4_D$ELOC <- (IE4_D$ELOC1+IE4_D$ELOC2)/2
describe(IE4_D$ILOC)
describe(IE4_D$ELOC)

#Internal locus of control
tapply(IE4_D$ILOC, IE4_D$SEX, describe)
AGE1_D <- subset(IE4_D, AGE == 18 | AGE == 19 | AGE == 20 | AGE == 21 | AGE == 22
| AGE == 23 | AGE == 24 | AGE == 25 | AGE == 26
        | AGE == 27 | AGE == 28 | AGE == 29)
AGE2_D <- subset(IE4_D, AGE == 30 | AGE == 31 | AGE == 32 | AGE == 33 | AGE == 34
| AGE == 35 | AGE == 36 | AGE == 37 | AGE == 38
        | AGE == 39 | AGE == 40 | AGE == 41 | AGE == 42 | AGE == 43 | AGE == 44 |
AGE == 45 | AGE == 46 | AGE == 47
        | AGE == 48 | AGE == 49)
AGE3_D <- subset(IE4_D, AGE == 50 | AGE == 51 | AGE == 52 | AGE == 53 | AGE == 54
| AGE == 55 | AGE == 56 | AGE == 57 | AGE == 58
```

```
	| AGE == 59 | AGE == 60 | AGE == 61 | AGE == 62 | AGE == 63 | AGE == 64 | AGE == 65 | AGE == 66 | AGE == 67
	| AGE == 68 | AGE == 69)

AGE1_D$ILOC <- (AGE1_D$ILOC1+AGE1_D$ILOC2)/2
describe(AGE1_D$ILOC)

AGE2_D$ILOC <- (AGE2_D$ILOC1+AGE2_D$ILOC2)/2
describe(AGE2_D$ILOC)

AGE3_D$ILOC <- (AGE3_D$ILOC1+AGE3_D$ILOC2)/2
describe(AGE3_D$ILOC)

#External locus of control
tapply(IE4_D$ELOC, IE4_D$SEX, describe)

AGE1_D$ELOC <- (AGE1_D$ELOC1+AGE1_D$ELOC2)/2
describe(AGE1_D$ELOC)

AGE2_D$ELOC <- (AGE2_D$ELOC1+AGE2_D$ELOC2)/2
describe(AGE2_D$ELOC)

AGE3_D$ELOC <- (AGE3_D$ELOC1+AGE3_D$ELOC2)/2
describe(AGE3_D$ELOC)

###############################################################################

#################################
#Step 7: Sample characteristics
#################################

##UK

#Age
describe(IE4_UK$age)

#Proportion of women
table(IE4_UK$sex)

#Educational level
table(IE4_UK$quota)
edu1 <- subset(IE4_UK, quota == 1 | quota == 2 | quota == 3 | quota == 10 |
	quota == 11 | quota == 12)
```

```
edu2 <- subset(IE4_UK, quota == 4 | quota == 5 | quota == 6 | quota == 13 |
        quota == 14 | quota == 15)
edu3 <- subset(IE4_UK, quota == 7 | quota == 8 | quota == 9 | quota == 16 |
        quota == 17 | quota == 18)
describe(edu1$quota)
describe(edu2$quota)
describe(edu3$quota)

##############################################################################

##Germany

#Age
describe(IE4_D$age)

#Proportion of women
table(IE4_D$sex)

#Educational level
table(IE4_D$quota)
edu1 <- subset(IE4_D, quota == 1 | quota == 2 | quota == 3 | quota == 10 |
        quota == 11 | quota == 12)
edu2 <- subset(IE4_D, quota == 4 | quota == 5 | quota == 6 | quota == 13 |
        quota == 14 | quota == 15)
edu3 <- subset(IE4_D, quota == 7 | quota == 8 | quota == 9 | quota == 16 |
        quota == 17 | quota == 18)
describe(edu1$quota)
describe(edu2$quota)
describe(edu3$quota)
```
